# Psychometric properties of the Burnout Assessment Tool - General version in nursing workers ^*^

**DOI:** 10.1590/1518-8345.7367.4425

**Published:** 2025-01-31

**Authors:** Lacir José Santin, Bianca Gonzalez Martins, Juliana Alvares Duarte Bonini Campos, Ana Claudia Souza Vazquez, Maria Helena Palucci Marziale, Isabel Amelia Costa Mendes, Neyson Pinheiro Freire, Wilmar B. Schaufeli, Hans De Witte, Fernanda Ludmilla Rossi Rocha

**Affiliations:** 1 Universidade de São Paulo, Escola de Enfermagem de Ribeirão Preto, PAHO/WHO Collaborating Centre for Nursing Research Development, Ribeirão Preto, SP, Brazil.; 2 Universidade Estadual Paulista, Faculdade de Ciências Farmacêuticas, Araraquara, SP, Brazil.; 3 Universidade Federal de Ciências da Saúde, Departamento de Psicologia, Porto Alegre, RS, Brazil.; 4 Conselho Federal de Enfermagem, Brasília, DF, Brazil.; 5 Katholieke Universiteit Leuven, Faculty of Psychology and Educational Sciences, Leuven, Belgium.; 6 Scholarship holder at the Coordenação de Aperfeiçoamento de Pessoal de Nível Superior (CAPES), Brazil.; 7 Scholarship holder at the Conselho Nacional de Desenvolvimento Científico e Tecnológico (CNPq), Brazil.

**Keywords:** Occupational Health, Nursing, Burnout, Psychometrics, Psychological Stress, Burnout Assessment Tool

## Abstract

**Objective:**

to analyze the validity evidence of the BAT – General version in a sample of Brazilian nursing workers.

**Method:**

a cross-sectional study design with non-probability sampling method was used among 3594 Brazilian nursing workers. The validity evidence was assessed by means of analysis based on the internal structure, on the relations to external variables, and on response process of the items.

**Results:**

the factor models of the BAT – General version showed goodness-of-fit to the data. However, the complete factor model enabled a better understanding of burnout syndrome in the sample. It was attested the BAT – General version dimensions were positively correlated with depression, anxiety and stress symptoms and negatively correlated with job satisfaction and satisfaction with life. The validity evidence analysis based on response process of the items revealed that BAT – General version works properly both in the group of nursing workers who reported having emotional or psychological health problems and in the group who denied these problems.

**Conclusion:**

the results provided robust validity evidence of the BAT – General version in Brazilian nursing workers.

## Highlights

**(1)** BAT – General version was shown to be a robust and consistent instrument.

**(2)** BAT – General version can become an important management tool for nursing leaders.

**(3)** It is a tool that can be used in the individual assessment of burnout symptoms.

**(4)** BAT – General version should not be used for clinic diagnosis of burnout.

## Introduction

Today, burnout stands out as one of the main psychological disorder related to chronic occupational stress. It was recently postulated by the World Health Organization as an occupational disease and included in the International Classification of Diseases, ICD-11^(1)^. Nursing is recognized as an essential profession for healthcare focused on individual needs^(2)^, whose work conditions and demands are directly related to the workers’ physical and mental illness, highlighting the burnout syndrome^(3-5)^. During the covid-19 pandemic, demand intensified, as did the precarious working conditions faced by nursing professionals at a global level, which contributed to the increased prevalence of burnout and other psychological problems among these workers^(6-8)^.

Despite the many definitions, it is a practically unanimous opinion that exhaustion represents the core component of burnout^(1,9-12)^. The most current and encompassing definition of burnout was proposed in 2020^(12)^ based on the set forth by the Job Demands–Resources (JD–R)^(13)^ modeland otherspecifics theoretical assumptions^(14)^. According to the JD–R model, burnout results from an imbalance between job demands (aspects that require physical or mental effort and that, in the long term, can culminate in worker exhaustion) and job resources (motivational factors and strategies workers use to deal with their work demands)^(15-16)^.

On the basis of these theoretical frameworks^(12)^, it was proposed a novel instrument, the Burnout Assessment Tool (BAT), considering burnout as a syndrome comprising four primary symptoms, i.e., exhaustion, mental distance, cognitive impairment, and emotional impairment, which can also be accompanied by two secondary symptoms, i.e., psychological distress and psychosomatic complaints^(12,17)^. According to the authors, exhaustion refers to severe energy loss that results in mental exhaustion, manifested as symptoms such as lacking the energy to start a new job; feeling completely worn out after an entire workday; feeling tired quickly, even after spending minimal effort at work; and being unable to relax after work. Mental distance represents psychological distancing from the job and a strong reluctance or aversion to work. The main characteristics of mental distance are indifference and cynical attitudes, with no interest in and enthusiasm for the job. Cognitive impairment refers to poor cognitive performance, memory problems, and attention and concentration deficits. Some specific symptoms are the difficulty to think clearly or learn new things at work, forgetfulness, absentmindedness, a lack of focus on work, and attention and concentration deficit. Emotional impairment involves feeling intense emotional reactions, irritable, frustrated and angry at work, and upset or sad without knowing why; being unable to control one’s emotions at work; and overreacting. The secondary symptoms related to psychological distress refer to psychological problems, such as sleep disorders; a feeling of tension, anxiety, or concern; and weight fluctuations. The psychosomatic complaints refer to physical symptoms that are exacerbated by or result from some psychological problems. Examples of such symptoms are palpitations, chest pain, stomach and intestinal problems, muscle pains, and headaches^(18)^.

Originally, the BAT was developed in two versions: the BAT – General version, with 32 context-free items and the BAT – Work-related version, with 33 items^(18)^. Basically, the difference between the general and the work-related versions is that, with the exception of the mental distance domain, the items from the BAT – General version refer to a person’s everyday life and not to the work context^(18)^. The BAT – Work-related version has been used at the global level and been already culturally adapted and validated in several cultural contexts^(19-25)^. To date, no previous studies have examined the psychometric properties of the BAT – General version among Brazilian nursing workers.

The validation studies of the BAT – Work-related version with samples comprising workers from different sectors of the economy have analyzed the adequacy of different factor models of the instrument: (1) the four primary and two secondary burnout symptoms are analyzed separately: BAT-C, primary symptoms, correlated 4-factor model; BAT-S, secondary symptoms, correlated 2-factor model; (2) the primary and secondary burnout symptoms are analyzed together: BAT32, four primary symptoms and two secondary symptoms, correlated 6-factor model; (3) BAT32 SOHM second-order hierarchical model (six first order and one second order construct – burnout). Examples are the studies conducted in several countries from Europe^(21,26-27)^, Asia^(23)^ and South America^(28)^, including Brazil^(17,24,29)^. The BAT – Work-related version was also applied in a sample comprising nursing staff members from Poland^(19)^ and among Italian healthcare workers^(27,30)^, demonstrating excellent psychometric properties in these contexts and populations.

Evidence have also pointed out that the primary symptoms of the BAT – Work-related version have positive correlations with job demands and negative correlations with job resources, supporting theoretical assumptions of the JD–R model^(13,31-32)^. Among the main job demands positively correlated with the primary burnout symptoms of the BAT are workload; turnover intention^(21,24-25)^; time pressure and role conflict^(21)^; workaholism or working excessively and compulsively^(23)^; negative affect^(19,24)^; high mental, emotional, and physical demands^(17,28)^; and bureaucracy, negative change, interpersonal conflicts^(17)^.

In relation to the job resources, negative correlations have been found between the primary burnout symptoms of the BAT and the following variables: work engagement^(19,21,28)^; job satisfaction^(19,21)^; autonomy, role clarity, and co-worker support^(21,24-25)^; vigor, dedication, and absorption^(23)^; organizational self-efficacy and self-esteem^(29)^; affective organizational commitment, optimism, social self-efficacy, and task self-efficacy^(21)^; job clarity, team support, supervisor, spirit perception, teamwork, and job control^(17,28)^; and perception about decision-making, and dispositional hope^(28)^.

Given the scarcity of studies, the aim of this investigation was to analyze the validity evidence of the BAT – General version in a sample of Brazilian nursing workers.

## Method

### Study design and sample

This is a cross-sectional study with non-probability sampling method. The population consisted of Brazilian nurses and nursing technicians and assistants. To calculate the minimum sample size required to perform the statistical analyses, the need for five to 10 respondents per parameter to be estimated in the model was considered^(33)^. Considering the 85 parameters of the BAT – General version (32 items, 32 errors, six latent factors, and 15 correlations between factors), the minimum sample size was calculated at between 425 and 850 participants. The following inclusion criteria were considered: being a nurse, nursing technician, or assistant and having an electronic address (e-mail) registered at the Brazilian Federal Nursing Council – COFEN; currently working in the profession; and having worked in the nursing area for at least one year.

The professionals were invited to participate in the study by means of an electronic message sent by the COFEN, which contained a link to access the data collection instruments available on the Research Electronic Data Capture (REDCap) platform. More recent data indicate a total of 2,727,473 registrations of nursing professionals in Brazil, of which 671,091 are nurses; 447,375 are technicians; and 1,608,653 are nursing assistants^(34)^. However, these data do not correspond to the total number of Brazilian nursing workers, which is due to the possibility of duplicate professional registrations. Actually, 779,337 email addresses are registered in the COFEN system, which corresponds to the total number of email messages sent in this study. In all, 5979 nursing workers voluntarily agreed to participate (adherence rate=0.77%) and 3594 completed questionnaires were identified, which were included in the sample (response rate=66.11%). Data collection took place between April 1 and July 31, 2022.

### Measures

Burnout was evaluated through the Portuguese Brazilian version of the Burnout Assessment Tool (BAT) – General version, which was culturally adapted and validated in Brazil by the authors of the original instrument themselves (available at https://burnoutassessmenttool.be/project_eng/). In the BAT – General version, the core symptoms comprise 22 items and assess four primary dimensions (exhaustion, mental distance, cognitive impairment, and emotional impairment); the secondary symptoms contain 10 items and assess two dimensions (psychological distress and psychosomatic complaints). The items are rated on a five-point Likert response scale (1=Never, 2=Rarely, 3=Sometimes, 4=Often times, and 5=Always)^(12)^. The global score of the instrument can be evaluated at four levels of burnout: low (< 25th percentile); moderate (25th percentile ┤ 75th percentile); high (75th percentile ┤ 95th percentile); and very high (> 95th percentile), based on the average score of participants’ responses^(18)^.

Satisfaction with life was assessed using the Portuguese Brazilian version of the Satisfaction with Life Scale (SWLS)^(35)^. The SWLS was originally developed in the English language to assess a person’s general perception in relation to satisfaction with life^(36)^ and has five items, which are answered using a seven-point Likert scale ranging from 1 (Strongly disagree) to 7 (Strongly agree). Evidence have proven excellent psychometric properties of the SWLS in different cultural contexts^(37-38)^.

Depression, anxiety, and stress symptoms were measured using the Portuguese Brazilian version of the Depression, Anxiety and Stress Scale (DASS-21)^(39)^. DASS-21 was originally developed in 1995^(40)^; it presents 21 items distributed in three dimensions: depression (items 3, 5, 10, 13, 16, 17, and 21), anxiety (items 2, 4, 7, 9, 15, 19, and 20), and stress (items 1, 6, 8, 11, 12, 14, and 18); and it uses a four-point Likert response scale ranging from 0 (It did not apply at all) to 3 (It applied a lot or most of the time)^(39)^. Several studies have shown the good psychometric properties of the DASS-21 comprising healthcare workers.

Job satisfaction was assessed by means of the following additional question in the participant characterization instrument: On a scale from 1 to 10, please indicate how satisfied you are with your job in nursing (1=very dissatisfied and 10=very satisfied)^(41-42)^.

### Procedures and statistical analysis

Following the recommendations of the Standards for Educational and Psychological Testing^(43)^, the psychometric properties of the BAT – General version was evaluated through validity evidence analysis based on the internal structure, on the relations to external variables, and on response process of the items.

The validity evidence based on the internal structure was conducted by means of factorial, convergent, and divergent construct validity. Factorial invariance and data reliability were also assessed. Factorial validity was tested using Confirmatory Factor Analysis (CFA), preceded by the psychometric sensitivity analysis of the data, considering the skewness (sk) and kurtosis (ku) absolute values less than 3 and 7, respectively, to ensure the assumption of the data normal distribution required by the Structural Equation Modeling method^(44)^. To perform the CFA, the robust Weighted Least Squares Means and Variance adjusted (WLSMV) method was used. To assess the quality of the models fit, a variety fit indices were measured: Comparative Fit index (CFI), Tucker–Lewis index (TLI), Root Mean Square Error of Approximation (RMSEA) with a 90% confidence interval (CI), and Standardized Root Mean Square Residual (SRMR), considered adequate if CFI and TLI>0.90, RMSEA<0.10, and SRMR<0.08^(45-46)^. The factor loadings (λ) of the BAT items were also evaluated and considered valid when λ≥0.50. Six alternative factor models of the BAT – General version were tested in the CFA: BAT-C (four correlated primary symptoms first-order factors, 22 items), BAT-S (two correlated secondary symptoms first-order factors, 10 items), BAT32-6 (six correlated primary and secondary symptoms first-order factors, 32 items), BAT32 SOHM (second-order hierarchical model, six correlated first-order factors and one second-order factor – burnout), BAT32-5 (four correlated primary symptoms first-order factors and one factor with 10 secondary symptoms, 32 items), and BAT12 (short version of four correlated primary symptoms first-order factors, 12 items)^(18)^.

Convergent validity of the factorial model was estimated by means of the Average Variance Extracted (AVE) of the BAT – General version factors, considered adequate if AVE≥0.50^(47)^. Discriminant validity of the factorial model was analyzed by comparing the AVE of each factor to the square of the correlation between the factors, considering that there is discriminant validity when the AVE of each factor is greater than or equal to the correlation squared between the factors (AVEi and AVEj≥ρij^2^)^(47)^. Factorial invariance of the model was tested between independent samples through the use of multigroup analysis and the CFI difference test (ΔCFI). Thus, the sample was randomly divided into two subgroups (test *n*=1799; validation *n*=1795) and the CFI values of the configural (M_0_), metric (M_1_), and scalar (M_2_) models were analyzed. ΔCFI values <0.01 (ΔCFI_M1-M0_ and ΔCFI_M2-M1_) were considered as indicative of factorial invariance^(45,48)^. Factorial invariance of the BAT – General version was also evaluated according to the presence or absence of emotional/psychological health problems (presence *n*=2500; absence *n*=1071) and according to gender. Considering the high discrepancy related to the number of male and female participants, a smaller subsample (20%) of women was randomly selected for comparison between groups (male *n*=484; female *n*=622). Data reliability was assessed using the ordinal alpha coefficient (α) and Composite Reliability (CR), considering α and CR values of ≥0.70 as indicative of suitable reliability^(47)^.

The validity evidence based on the relations to external variables was evaluated using the convergent validity of the latent correlations of the BAT – General version dimensions with personal/occupational demands and resources. Burnout and its primary and secondary symptoms were expected to show positive correlations with depression, anxiety and stress (demands) and negative correlations with satisfaction with life and job satisfaction (resources). The Portuguese Brazilian version of the Depression, Anxiety and Stress Scale (DASS-21)^(39)^ was used to estimate the correlation between burnout symptoms and depression, anxiety, and stress and presented adequate fit to the sample [λ=0.64-0.90, TLI=0.968, CFI=0.971, RMSEA=0.078 (90%CI=0.076–0.080), SRMR=0.036, and α=0.92-0.94]. To evaluate the correlation between burnout symptoms and general satisfaction with life, we used the Portuguese Brazilian version of the Satisfaction with Life Scale (SWLS)^(35)^, which also presented adequate adjustment to the sample [λ=0.69-0.89, TLI=0.990, CFI=0.995, RMSEA=0.092 (90%CI=0.080-0.104), SRMR=0.017, and α=0.89].

Validity based on response process of the items was evaluated using the Differential Item Functioning (DIF) analysis between two distinct sample subgroups: presence (*n*=2500) of emotional/psychological health problems and absence (*n*=1071) of emotional/psychological problems. The classification of these groups occurred based on the answer to the following question presented in the sample characterization instrument: “In the last 30 days, have you worked with any emotional/psychological problem?”, the answer to which was dichotomous (yes and no).

DIF analysis was conducted using ordinal logistic regression based on the likelihood ratio chi-square statistics (significance level of 1%) and considering the Partial-Credit Model (PCM). The indices Information-Weighted Mean Square (Infit) and the Unweighted Mean Square (Outfit) of the complete BAT – General version factor model (BAT32-6) were also estimated. Infit and Outfit values between 0.5 and 1.5 indicated adequate fit of the item to the PCM^(49-50)^. To assess the effect size of the DIF analysis, McFadden’s and Negelkerke’s pseudo R^2^ coefficients were used, with R^2^<0.13^(51)^ being considered negligible; items that presented a significant total DIF effect (*p*<0.01) were considered non-equivalent.

For statistical analyses, the IBM SPSS Statistics 22 (IBM Corp., Armonk, N.Y., USA) and R programs^(50)^ with lavaan (version 0.6-10) ^(52)^, SemTools (version 0.5-5)^(53)^, lordif^(51)^ and eRm packages were used.

## Results

The majority of participants were female (n=3090; 85.98%); 1789 (49.78%) were married; 3006 (83.64%) were aged 19-44 years (mean age=35.75; SD=10.09); 1722 (47.91%) were nurses and 1862 (51.81%) were nursing technicians or assistants by profession; 2468 (68.67%) had one to 10 years of professional experience; 1823 (50.72%) were working at hospital services and 847 (23.57%) at primary healthcare units.

In the validity evidence analysis based on the internal structure, the psychometric sensitivity showed adequate values of skewness (sk=-0.49–1.42) and kurtosis (ku=-1.30–1.81), proving the normal distribution of the data. The Confirmatory Factor Analysis (CFA) and the reliability analysis of six alternative BAT – General version factor models are presented in Table 1.

**Table 1:** - Confirmatory Factor Analysis and reliability of alternative BAT – General version models (n = 3594). Brazil, 2024

**Model**	λ*	**TLI** ^†^	**CFI** ^‡^	**RMSEA** ^§^ **[90%CI]** ^||^	**SRMR** ^¶^	AVE**	**CR** ^††^	**α** ^‡‡^
BAT-C^§§^	0.768-0.927	0.964	0.969	0.092 [0.090-0.094]	0.040	0.732-0.769	0.916-0.964	0.910-0.961
BAT-S^||||^	0.719-0.920	0.972	0.979	0.099 [0.094-0.104]	0.039	0.608-0.661	0.886-0.906	0.881-0.897
BAT32-6^¶¶^	0.711-0.940	0.961	0.965	0.073 [0.071-0.074]	0.035	0.609-0.769	0.886-0.964	0.881-0.961
BAT32-5***	0.706-0.937	0.957	0.961	0.077 [0.075-0.078]	0.039	0.590-0.769	0.916-0.964	0.910-0.961
BAT12^†††^	0.790-0.924	0.981	0.986	0.084 [0.080-0.088]	0.032	0.704-0.824	0.877-0.934	0.872-0.931
BAT32 SOHM^‡‡‡^	0.710-0.941	0.955	0.959	0.078 [0.077-0.079]	0.044	0.609-0.769	0.886-0.964	0.881-0.961

*λ = Factor loadings; ^†^TLI = Tucker-Lewis index; ^‡^CFI = Comparative Fit Index; ^§^RMSEA = Root Mean Square Error of Approximation; ^||^90% CI = 90% Confidence Interval; ^¶^SRMR = Standardized Root Mean Square Residual; **AVE = Average variance extracted; ^††^CR = Composite reliability; ^‡‡^α = Ordinal alpha coefficient. ^§§^BAT-C = Core/primary symptoms; ^||||^BAT-S = Secondary symptoms; ^¶¶^BAT32-6 = Primary and secondary symptoms; ***BAT32-5 = Primary symptoms and 1 factor with 10 secondary symptoms; ^†††^BAT12 = Short version of primary symptoms; ^‡‡‡^BAT32 SOHM = Second-order hierarchical model

The results confirmed the goodness-of-fit of all tested models. However, the complete factor model of the instrument (BAT32-6) presented the best fit to the data [λ=0.711-0.940; TLI=0.961; CFI=0.965; RMSEA=0.073 (90%CI=0.071-0.074); SRMR=0.035] and it was preferred for further analysis.

The convergent validity of the BAT32-6 factors was attested (AVE=0.609–0.769); however, it was not possible to confirm the discriminant validity between the factors psychological distress and exhaustion (r^2^=0.716, *p*<0.001); psychological distress and emotional impairment (r^2^=0.719, *p*<0.001); psychological distress and psychosomatic complaints (r^2^=0.740, *p*<0.001); psychosomatic complaints and exhaustion (r^2^=0.616, *p*<0.001), due to the strong correlations between them. The reliability of the BAT32-6 factors was also adequate.

Regarding the validity evidence based on the relations to external measures, Table 2 presents the correlation between the BAT32-6 factors and external variables. It was confirmed that burnout and all the BAT32-6 factors were positively correlated with the symptoms of depression, anxiety and stress (demands) and negatively correlated with satisfaction with life and job satisfaction (resources), as expected, supporting theoretical assumptions of the JD–R model.

**Table 2:** - Correlations between the BAT32-6 factors and external variables (n = 3594). Brazil, 2024

	EX*	**MD** ^†^	**CI** ^‡^	**EI** ^§^	**PD** ^||^	**PC** ^¶^	BURNOUT**
**DEPR** ^††^	0.779	0.751	0.584	0.771	0.774	0.671	0.849
**ANX** ^‡‡^	0.711	0.636	0.534	0.723	0.782	0.789	0.812
**STRES** ^§§^	0.779	0.676	0.606	0.812	0.827	0.706	0.865
**SWLS** ^||||^	-0.472	-0.455	-0.347	-0.432	-0.459	-0.418	-0.505
**JOBSATISF** ^¶¶^	-0.492	-0.623	-0.376	-0.421	-0.442	-0.395	-0.529

*EX = Exhaustion; †MD = Mental distance; ^‡^CI = Cognitive impairment; ^§^EI = Emotional impairment; ^||^PD = Psychological distress; ^¶^PC = Psychosomatic complaints; **BURNOUT = Second-order hierarchical model; ^††^DEPR = Depression; ^‡‡^ANX = Anxiety; ^§§^STRES = Stress; ^||||^SWLS = Satisfaction with life; ^¶¶^JOBSATISF = Job satisfaction. All the correlations presented *p*<0.01

The BAT32-6 invariance analysis in independent subgroups (test and validation) and according to the occurrence of emotional/psychological health problems (presence and absence) is presented in Table 3. Strong/strict measure invariance was observed between configural, metric, and scalar models of the different subgroups, reinforcing the internal consistency of the instrument.

**Table 3:** - Invariance analysis of six correlated primary and secondary symptoms first-order factors, 32 items (BAT32-6) in different groups (n = 3594). Brazil, 2024

**Samples**	**n**	λ*	**TLI** ^†^	**CFI** ^‡^	**RMSEA** ^§^ **[90%CI]** ^||^	**SRMR** ^¶^	α**
Test	1799	0.714-0.945	0.964	0.967	0.070 [0.068-0.072]	0.036	0.879-0.961
Validation	1795	0.708-0.935	0.962	0.965	0.072 [0.070-0.074]	0.037	0.883-0.961
Invariance (CFI)^¶^ M_0_:Configural=0.966; M_1_:Metric=0.967; M_2_: Scalar=0.968; ∆CFI<0.01 (M_1_-M_0_; M_2_-M_1_)							
Male	484	0.763-0.948	0.967	0.970	0.070 [0.066-0.074]	0.041	0.913-0.963
Female	622	0.727-0.945	0.967	0.970	0.065 [0.062-0.069]	0.040	0.877-0.962
Invariance (CFI)^¶^ M_0_:Configural=0.970; M_1_: Metric=0.970; M_2_: Scalar=0.969; ∆CFI<0.01 (M_1_-M_0_; M_2_-M_1_)							
+ Emotional problems^††^	2500	0.654-0.926	0.950	0.955	0.072 [0.071-0.074]	0.040	0.854-0.950
- Emotional problems^‡‡^	1071	0.685-0.929	0.959	0.963	0.065 [0.063-0.068]	0.043	0.867-0.956
Invariance (CFI)^¶^ M_0_: Configural=0.957; M_1_: Metric=0.957; M_2_: Scalar=0.960; ∆CFI<0.01 (M_1_-M_0_; M_2_-M_1_)							

*λ = Factor loadings; ^†^TLI = Tucker-Lewis index; ^‡^CFI = Comparative fit index; ^§^RMSEA = Root mean square error of approximation; ^||^90%CI = 90% Confidence Interval; ^¶^SRMR = Standardized root mean square residual; **α = Ordinal alpha coefficient; ^††^+ Emotional problems = Presence of emotional/psychological health problems; ^‡‡^- Emotional problems = Absence of emotional/psychological health problems

It is also important to highlight the CFA of the BAT32 SOHM, which proved that burnout was strongly reflected by all factors (β=0.769–0.942, *p*<0.001), specially by psychological distress (β=0.942, *p*<0.001), exhaustion (β=0.888, *p*<0.001), and emotional impairment (β=0.891, *p*<0.001).

In relation to the validity evidence based on response process of the items, Table 4 presents the fit indices of the BAT32-6 (Infit and Outfit values) and the DIF analysis results between sample subgroups (presence or absence of emotional/psychological health problems).

**Table 4:** - Fit indices and differential item functioning analysis between sample subgroups (n = 3594). Brazil, 2024

**Item**	Subgroup 1*		**Subgroup 2** ^†^		**DIF**	**Pseudo R^2^**	
	**Infit**	**Outfit**	**Infit**	**Outfit**	p-**value**	**McFadden**	**Nagelkerke**
1	0.803	0.800	0.820	0.819	0.000	0.002	0.002
2	0.942	0.946	1.065	1.173	0.079	0.001	0.001
3	0.788	0.788	0.777	0.779	0.039	0.001	0.001
4	0.815	0.793	0.846	0.848	0.370	0.000	0.000
5	0.792	0.777	0.797	0.770	0.625	0.000	0.000
6	0.775	0.760	0.702	0.655	0.917	0.000	0.000
7	0.830	0.816	0.849	0.842	0.004	0.001	0.001
8	0.771	0.753	0.776	0.767	0.329	0.000	0.000
9	0.909	0.910	1.031	1.058	0.199	0.000	0.000
10	1.090	1.229	1.252	2.308	0.199	0.000	0.000
11	1.031	1.071	1.053	1.031	0.953	0.000	0.000
12	1.171	1.308	1.205	1.675	0.103	0.000	0.001
13	0.942	0.927	0.938	0.895	0.058	0.001	0.001
14	1.075	1.117	1.183	1.391	0.013	0.001	0.001
15	1.110	1.122	1.105	1.150	0.000	0.003	0.005
16	0.985	0.974	1.026	1.018	0.000	0.002	0.003
17	1.137	1.079	1.062	1.093	0.003	0.001	0.003
18	0.841	0.820	0.840	0.812	0.838	0.000	0.000
19	0.873	0.853	0.746	0.659	0.322	0.000	0.000
20	1.097	1.112	1.027	1.055	0.202	0.000	0.001
21	0.764	0.768	0.731	0.698	0.003	0.001	0.001
22	0.895	0.914	0.852	0.827	0.892	0.000	0.000
23	1.276	1.369	1.233	1.634	0.068	0.001	0.001
24	0.904	0.929	0.937	0.959	0.175	0.000	0.000
25	0.692	0.691	0.680	0.683	0.000	0.002	0.001
26	1.000	1.007	0.944	0.936	0.000	0.003	0.005
27	1.150	1.273	1.227	1.382	0.105	0.000	0.001
28	1.172	1.210	1.138	1.227	0.001	0.002	0.003
29	1.263	1.372	1.185	1.249	0.359	0.000	0.000
30	1.243	1.324	1.209	1.233	0.118	0.000	0.001
31	1.163	1.214	1.198	1.225	0.186	0.000	0.001
32	1.099	1.118	1.018	0.954	0.139	0.000	0.001

*Subgroup 1 = Presence of emotional/psychological health problems; ^†^Subgroup 2 = Absence of emotional/psychological health problems

The DIF analysis results indicated that in the subgroup 1 (presence of emotional/psychological health problems), all of the BAT32-6 items displayed adequate fit to the PCM (Infit and Outfit values between 0.5 and 1.5). In the subgroup 2 (absence of emotional/psychological problems), the items 10, 12 e 23 presented Outfit values > 1.5 (inadequate fit to the PCM). In addition, the items 1, 7, 14, 15, 16, 17, 21, 25, 26 e 28 were considered non-equivalent (*p*<0.01), indicating that they were answered differently by the participants of each subgroup. However, the effect size of the DIF in both groups was considered negligible for all items (R^2^<0.13).

**Figure 1:**
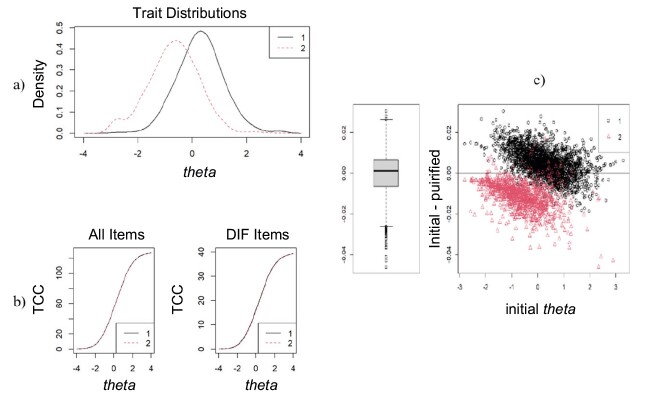
- (a) latent trait distribution of the participants; (b) Items Characteristic Curves – for all items and just for items having DIF; (c) boxplot of latent trait distribution in both subgroups (n = 3594). Brazil, 2024

The Figure 1 shows the latent trait of the participants and the Items Characteristic Curve (ICC) in both subgroups (presence and absence of emotional/psychological health problems).

The latent trait distribution in each subgroup (Figures 1a and 1c) showed a higher level of theta (θ) among participants who reported having emotional/psychological health problems (subgroup 1) and indicate that the instrument seems to properly discriminate individuals with and without emotional health problems. The ICC (Figure 1b) were found to be coincident when the items are analyzed together, confirming the invariance of the model between the subgroups.

## Discussion

This study confirmed the excellent psychometric properties of the BAT – General version applied to a sample of Brazilian nursing workers.

In the validity evidence analysis based on the internal structure, the results of the CFA supported the goodness-of-fit of all factorial models of the BAT – General version in the sample: complete model, 32 items and six correlated factors (BAT32-6); 23 items and the four correlated primary symptoms model (BAT-C); second-order hierarchical model (BAT32 SOHM); and short version, 12 items (BAT12). However, the BAT32-6 represented the most robust alternative for assessing burnout symptoms among the Brazilian nursing workers.

These results corroborate validation studies of the BAT – Work-related version that have confirmed the psychometric properties of first and second-order BAT-C^(24,28,54-55)^ models and the validity of the short version (BAT12)^(21,25)^, reinforcing the possibility of using the 12-items version to evaluate burnout symptoms in different occupational and clinical contexts. The choice to use the complete or the short form of the BAT – General version will depend on the health service context and the purpose of use (a quick screening, monitoring, or follow-up of nursing workers’ health symptoms).

The correlations between the BAT32-6 dimensions attested to convergent construct validity and are in line with the instrument’s theoretical assumptions, which consider the burnout as a syndrome characterized by four interrelated dimensions of primary symptoms and two dimensions of secondary symptoms^(18)^.

Regarding the validity analysis based on the relations to external variables, the results showed positive correlations between symptoms of burnout, depression, anxiety, and stress (demands) and negative correlations between burnout symptoms and the satisfaction with life and the job satisfaction constructs (resources), confirming the assumptions of the job demands–resources (JD–R) model as a conceptual framework. According to the JD–R model, job demands represent individual, social, or organizational aspects of the job that require constant physical or mental effort (stressors) and lead, in the long term, to the burnout syndrome; job resources refer to physical, psychological, social, or organizational aspects of the job capable of (a) reducing the physiological and psychological costs associated with the job demands, (b) helping workers achieve work goals, and (c) stimulating personal growth and development^(13,56)^. Therefore, job resources are understood as health-protecting factors with a mediating role of utmost importance to mitigate the impacts of job demands and to predict motivation, engagement, and satisfaction at work^(57)^.

Theoretical understanding of the work-related health and illness process from the perspective of a balance between demands and resources related to individual and occupational aspects represents the main reason for the increasing use and improvement of the JD–R^(15)^ model. Over the years, characteristics intrinsic to the subject itself, such as satisfaction with life^(17)^, work engagement^(56)^, and resilience^(58)^ have been recognized as forms of resourcess^(16)^.

In this sense, the negative correlations between satisfaction with life (measured through the Satisfaction with Life Scale) and the symptoms of burnout, depression, anxiety, and stress are highlighted. Satisfaction with life can be understood as a global perception of one’s own life experience^(59)^, and it is frequently associated with well-being and recognized as a protective factor against psychological symptoms^(60)^. These results corroborate a validation study of the BAT – Work-related version that theoretically discusses the relationship between these constructs and the mediating role of satisfaction with life in relation to burnout^(17)^. Among nurses, negative correlations between satisfaction with life and symptoms of depression, anxiety, and stress was also attested^(17,61)^.

In this regard, it is important to highlight the characteristics of the social context and the participants of this study, that is, nursing workers who experienced all the difficulties related to facing the covid-19 pandemic while acting on the front line of care and who still suffer the consequences of this health crisis in Brazil. These social and occupational conditions may have seriously affected general satisfaction with life of this professional group.

The positive correlations attested between symptoms of burnout, depression, anxiety, and stress corroborate evidence from studies carried out among healthcare professionals in Brazil^(62)^, Istanbul^(63)^, and Portugal^(64)^, which attested positive correlations between depression and burnout and negative correlations between exhaustion, depression, and resilience, suggesting the mediating role of resilience in the occurrence of these symptoms. From the JD–R perspective, resilience represents an individual resource for nursing workers^(58)^.

However, in these studies, the BAT was not used to evaluate the relation between symptoms of burnout, depression, anxiety, and stress; other psychometric instruments, such as the Copenhagen Burnout Inventory^(63-64)^ and the Maslach Burnout Inventory^(62-63)^ were used. This fact reveals an important weakness in the comparison of results and, at the same time, reasserts the originality of this study since, to the present day, no evidence has been found on the concomitant use of the BAT and DASS-21 among healthcare workers.

Regarding the negative correlations between the latent variables investigated, it corroborates the findings of BAT – Work-related^(21,24-25,28)^ version validation studies related to burnout symptoms and job satisfaction^(3)^, as well as the scientific evidence regarding the relationships between work satisfaction and depression, anxiety, and stress symptoms^(65-66)^.

In relation to the validity evidence based on response process of the items, the results showed that BAT32-6 works properly in both subgroup of nursing workers, those who reported having and those who denied emotional/psychological health problems, indicating that the BAT – General version can be used both in the normative population and among individuals affected by psychological problems.

Finally, it’s highlighted the main scientific contribution of this manuscript lies in the presentation of a new valid and reliable instrument for evaluating burnout symptoms in Brazilian nursing workers, which has a robust and updated theoretical framework, in line with recent transformations that have occurred in workplaces globally.

About the limitations of this study, the scarcity of validation studies of the BAT – General version to this day stands out, which precludes an in-depth analysis and comparing results. On the other hand, it reinforces the originality and relevance of this investigation.

## Conclusion

The BAT – General version was shown to be a robust and consistent instrument for measuring burnout symptoms in Brazilian nursing workers. Therefore, the BAT – General version can become an important management tool for nursing leaders and other healthcare managers who seek to promote healthy workplaces, covering physical, psychological, and emotional workers’ health aspects.

In the end, it is noted that the original authors advise that the BAT should not be used for clinical diagnosis of burnout. Instead, it is a valuable measuring instrument to be used in the assessment of the individual’s level of burnout symptoms.
