# Supplementary material for: Psychometric properties of the Burnout Assessment Tool - General version in nursing workers
Source: Rev Lat Am Enfermagem. 2025 Jan 31;33:e4425. doi: 10.1590/1518-8345.7367.4425 (PMC11789741; doi:10.1590/1518-8345.7367.4425)
Supplement: Translation

# Translation: Propiedades psicométricas del *Burnout Assessment Tool* – Versión general en trabajadores de enfermería^*^

**DOI:** 10.1590/1518-8345.7367.4424

**Authors:** Lacir José Santin, Bianca Gonzalez Martins, Juliana Alvares Duarte Bonini Campos, Ana Claudia Souza Vazquez, Maria Helena Palucci Marziale, Isabel Amelia Costa Mendes, Neyson Pinheiro Freire, Wilmar B. Schaufeli, Hans De Witte, Fernanda Ludmilla Rossi Rocha

**Affiliations:** 1 Universidade de São Paulo, Escola de Enfermagem de Ribeirão Preto, PAHO/WHO Collaborating Centre for Nursing Research Development, Ribeirão Preto, SP, Brasil.; 2 Universidade Estadual Paulista, Faculdade de Ciências Farmacêuticas, Araraquara, SP, Brasil.; 3 Universidade Federal de Ciências da Saúde, Departamento de Psicologia, Porto Alegre, RS, Brasil.; 4 Conselho Federal de Enfermagem, Brasília, DF, Brasil.; 5 Katholieke Universiteit Leuven, Faculty of Psychology and Educational Sciences, Leuven, Bélgica.; 6 Becaria de la Coordenação de Aperfeiçoamento de Pessoal de Nível Superior (CAPES), Brasil.; 7 Becaria del Conselho Nacional de Desenvolvimento Científico e Tecnológico (CNPq), Brasil.

**Keywords:** Salud Laboral, Enfermería, Burnout, Psicometría, Agotamiento Psicológico, Burnout Assessment Tool

## Abstract

**Objetivo:**

analizar la evidencia de validez del BAT – Versión general en una muestra de trabajadores de enfermería brasileños.

**Método:**

se realizó un estudio transversal, con muestreo no probabilístico, con 3594 trabajadores de enfermería brasileños. La validez del instrumento se evaluó mediante análisis basado en la estructura interna, basado en las relaciones con variables externas y basado en el proceso de respuesta a los ítems.

**Resultados:**

los modelos factoriales del BAT – Versión general mostraron ajuste adecuado a los datos. Sin embargo, el modelo factorial completo posibilitó una mejor comprensión del síndrome de *burnout* en la muestra. Se comprobó que los factores del BAT – Versión general presentaron correlaciones positivas con síntomas de depresión, ansiedad y estrés y correlaciones negativas con la satisfacción laboral y la satisfacción general con la vida. El análisis de las evidencias de validez basado en el proceso de respuesta a los ítems reveló que el BAT – Versión general funcionó adecuadamente en ambos grupos de trabajadores de enfermería, los que informaron que tenían problemas de salud emocional o psicológica y los que lo negaron.

**Conclusión:**

los resultados proporcionaron evidencia sólida de la validez del BAT – Versión general en trabajadores de enfermería brasileños.

## Destacados

**(1)** Se demostró que el BAT – Versión general es un instrumento robusto y consistente.

**(2)** El BAT – Versión general puede convertirse en una importante herramienta de gestión para los líderes de enfermería.

**(3)** Es una herramienta que se puede utilizar en la evaluación individual de los síntomas de *burnout*.

**(4)** No hay que utilizar el BAT – Versión general para el diagnóstico clínico de *burnout*.

## Introducción

Actualmente, el *burnout* se destaca como uno de los principales trastornos psicológicos relacionados con el estrés laboral crónico. Recientemente, la Organización Mundial de la Salud lo postuló como una enfermedad profesional y la incluyó en la Clasificación Internacional de Enfermedades, CIE-11^(1)^. La enfermería es reconocida como una profesión esencial para la atención médica centrada en las necesidades individuales^(2)^, cuyas condiciones y exigencias de trabajo se relacionan directamente con las enfermedades físicas y mentales de los trabajadores, entre las que se destaca el síndrome de *burnout* ^(3-5)^. Durante la pandemia de covid-19, la demanda se intensificó, al igual que las condiciones laborales precarias que enfrentan los profesionales de enfermería a nivel mundial, lo que contribuyó al aumento de la prevalencia de *burnout* y otros problemas psicológicos en dichos trabajadores^(6-8)^.

Pese a las numerosas definiciones que hay de *burnout*, es prácticamente unánime que el agotamiento representa el componente central del mismo^(1,9-12)^. La definición más actual y completa de *burnout* se propuso en 2020^(12)^ y se basa en el modelo *Job Demands–Resources* (JD-R)^(13)^ y en otros supuestos teóricos específicos^(14)^. Según el modelo JD-R, el *burnout* resulta de un desequilibrio entre las demandas laborales (aspectos que requieren esfuerzo físico o mental y que, a largo plazo, pueden culminar en el agotamiento del trabajador) y los recursos laborales (factores y estrategias motivacionales que los trabajadores utilizan para lidiar con las demandas laborales)^(15-16)^.

A partir de esas estructuras teóricas^(12)^, se propuso un nuevo instrumento para evaluar el *burnout*, el *Burnout Assessment Tool* (BAT), que considera el *burnout* como un síndrome compuesto por cuatro síntomas primarios (agotamiento, distancia mental, deterioro mental y deterioro cognitivo), que pueden ir acompañados de dos síntomas secundarios (*distress* psicológico y quejas psicosomáticas)^(12,17)^. Según los autores, el agotamiento se refiere a la pérdida severa de energía que genera agotamiento mental, manifestado por síntomas como falta de energía para iniciar un nuevo trabajo; sentirse completamente agotado después de un día entero de trabajo; sentirse cansado rápidamente, incluso después de un mínimo esfuerzo en el trabajo; e incapacidad para relajarse después del trabajo. La distancia mental representa un distanciamiento psicológico del trabajo y un fuerte desgano o aversión al trabajo. Las principales características de la distancia mental son la indiferencia, las actitudes cínicas y la falta de interés o entusiasmo por el trabajo. El deterioro mental implica rendimiento cognitivo deficiente, problemas de memoria y déficits de atención y concentración. Algunos síntomas específicos son dificultad para pensar con claridad o aprender cosas nuevas en el trabajo, olvidos, distracciones, falta de concentración en el trabajo. El deterioro cognitivo se manifiesta mediante reacciones emocionales intensas, irritabilidad, frustración y enojo en el trabajo, sentirse molesto o triste sin saber por qué; incapacidad para controlar las emociones en el trabajo; y reacciones exageradas. Los síntomas secundarios relacionados con el *distress* psicológico se refieren a problemas psicológicos como tener alteraciones del sueño, tensión, ansiedad o preocupación y fluctuaciones de peso. Las quejas psicosomáticas se refieren a síntomas físicos exacerbados o provocados por problemas psicológicos. Algunos ejemplos de esos síntomas son palpitaciones, dolor en el pecho, problemas gástricos e intestinales, dolores musculares y cefaleas^(18)^.

Originalmente, se desarrollaron dos versiones del BAT: el BAT – Versión general, con 32 ítems sin contexto, y el BAT – Relacionado con el trabajo, con 33 ítems^(18)^. Básicamente, la diferencia entre la versión general y la relacionada con el trabajo es que, a excepción del dominio distancia mental, los ítems del BAT – Versión general hacen referencia a la vida cotidiana del individuo y no al contexto laboral^(18)^. El BAT – Relacionado con el trabajo se ha utilizado mundialmente y fue adaptado culturalmente y validado en diversos contextos culturales^(19-25)^. Hasta ahora, no se encontró ningún estudio que analizara las propiedades psicométricas del BAT – Versión general en trabajadores de enfermería brasileños.

Los estudios de validación del BAT – Relacionado con el trabajo en muestras que incluyen trabajadores de diferentes sectores de la economía analizaron la adecuación de diferentes modelos factoriales del instrumento: (1) se analizan por separado los cuatro síntomas primarios y dos síntomas secundarios del *burnout*: BAT- C, modelo compuesto por los cuatro factores que representan los síntomas primarios del *burnout*, correlacionados; BAT-S, modelo compuesto por los dos factores que representan síntomas secundarios correlacionados; (2) se analizan conjuntamente los síntomas primarios y secundarios del *burnout*: BAT32, modelo completo compuesto por seis factores, representados por los cuatro síntomas primarios y los dos síntomas secundarios del *burnout*, correlacionados; (3) modelo jerárquico de segundo orden (MJSO) del BAT32 (seis factores de primer orden y un constructo de segundo orden - *burnout*). Ejemplos de ello son estudios realizados en varios países de Europa^(21,26-27)^, Asia^(23)^ y América del Sur^(28)^, incluido Brasil^(17,24,29)^. El BAT – Relacionado con el trabajo también se aplicó a una muestra compuesta por miembros del equipo de enfermería de Polonia^(19)^ y a profesionales de la salud italianos^(27,30)^, y demostró que tiene excelentes propiedades psicométricas en esos contextos y poblaciones.

La evidencia también indica que los síntomas primarios del BAT – Relacionado con el trabajo presentan correlaciones positivas con las demandas laborales y negativas con los recursos laborales, lo que ratifica los presupuestos teóricos del modelo JD-R^(13,31-32)^. Entre las principales demandas laborales que presentan correlaciones positivas con el BAT se encuentran: sobrecarga de trabajo, intención de abandonar el empleo o la profesión^(21,24-25)^, presión del tiempo y conflicto de funciones^(21)^, adicción al trabajo (trabajo excesivo y compulsivo)^(23)^, afecto negativo^(19,24)^, altas exigencias mentales, emocionales y físicas^(17,28)^, burocracia, cambios negativos y conflictos interpersonales^(17)^.

En cuanto a los recursos laborales, se encontraron correlaciones negativas entre los síntomas primarios del BAT y las siguientes variables: compromiso laboral^(19,21,28)^; satisfacción laboral^(19,21)^; autonomía, claridad de funciones y apoyo de los compañeros de trabajo^(21,24-25)^; vigor, dedicación y asimilación/concentración mental^(23)^; autoeficacia organizacional y autoestima^(29)^; compromiso organizacional afectivo, optimismo, autoeficacia social y autoeficacia en la realización de tareas^(21)^; claridad en el desarrollo de las actividades laborales, apoyo del equipo, supervisión, percepción espiritual, trabajo en equipo y control del trabajo^(17,28)^; y percepción sobre la toma de decisiones y esperanza disposicional^(28)^.

Dada la escasez de estudios, el objetivo de esta investigación es analizar las evidencias de validez del BAT – Versión general en una muestra de trabajadores de enfermería brasileños.

## Método

### Diseño del estudio y muestra

Estudio transversal con muestreo no probabilístico. La población estuvo compuesta por enfermeros, técnicos y auxiliares de enfermería brasileños. Para calcular el tamaño mínimo de la muestra necesario para realizar el análisis estadístico, se consideró que era necesario contar con de cinco a diez entrevistados por cada uno de los parámetros del modelo que se va a evaluar^(33)^. Considerando los 85 parámetros del BAT – Versión general (32 ítems, 32 errores, seis factores latentes y 15 correlaciones entre los factores), se calculó que el tamaño mínimo de la muestra debía estar entre 425 y 850 participantes. Se consideraron los siguientes criterios de inclusión: ser enfermero, técnico o auxiliar de enfermería y tener dirección de correo electrónico (e-mail) registrada en el Consejo Federal de Enfermería de Brasil (COFEN); ejercer actualmente la profesión; y haber trabajado en el área de enfermería durante al menos un año.

Los profesionales fueron invitados a participar a través de un mensaje electrónico enviado por el COFEN, que contenía un *link* para acceder a los instrumentos de recolección de datos disponibles en la plataforma electrónica *Research Electronic Data Capture* (REDCap). Los datos más recientes del COFEN indican que hay en total 2.727.473 de registros de profesionales de enfermería en Brasil, de los cuales 671.091 son enfermeros; 447.375 son técnicos; y 1.608.653 son auxiliares de enfermería^(34)^. Sin embargo, esos datos no coinciden con el número total de trabajadores de enfermería brasileños, lo que se debe a que, probablemente, haya registros profesionales duplicados. Actualmente, hay 779.337 direcciones de correo electrónico registradas en el sistema COFEN, lo que corresponde al número total de mensajes de correo electrónico enviados en este estudio. En total, 5979 profesionales de enfermería aceptaron participar voluntariamente (tasa de adherencia=0,77%) y se identificaron 3594 instrumentos cumplimentados completamente, que fueron los que conformaron la muestra (tasa de respuesta=66,11%). La recolección de datos se realizó entre el 1 de abril y el 31 de julio de 2022.

### Medidas

El *burnout* se evaluó utilizando la versión brasileña del *Burnout Assessment Tool* (BAT) - Versión general, instrumento que fue adaptado culturalmente y validado para el contexto brasileño por los autores del instrumento original (disponible en https://burnoutassessmenttool.be/project_eng/). En el BAT – Versión general, los síntomas centrales comprenden 22 ítems y evalúan cuatro dimensiones principales (agotamiento, distancia mental, deterioro mental y deterioro cognitivo); los síntomas secundarios tienen 10 ítems y evalúan dos dimensiones (*distress* psicológico y quejas psicosomáticas). Las opciones de respuesta de los ítems corresponden a una escala Likert de cinco puntos (1=Nunca, 2=Raramente; 3=Algunas veces; 4=A menudo; 5=Siempre)^(12)^. El puntaje total del instrumento se puede evaluar en cuatro niveles de *burnout*: bajo (< 25 percentil); moderado (de 25 a 75 percentil); alto (de 75 a 95 percentil); y muy alto (> 95 percentil), según la puntuación media de las respuestas de los participantes^(18)^.

La satisfacción con la vida fue evaluada mediante la versión brasileña de la *Satisfaction with Life Scale* (SWLS)^(35)^. La SWLS se desarrolló originalmente en inglés para evaluar la percepción general del individuo sobre la satisfacción con la vida^(36)^ y tiene cinco ítems, que se responden mediante una escala tipo Likert de siete puntos que van desde 1 (muy en desacuerdo) a 7 (totalmente de acuerdo). La evidencia prueba que la SWLS tiene excelentes propiedades psicométricas en diferentes contextos culturales^(37-38)^.

Los síntomas de depresión, ansiedad y estrés se evaluaron mediante la *Depression, Anxiety and Stress Scale* (DASS-21)^(39)^, que se desarrolló originalmente en 1995^(40)^ y presenta 21 ítems distribuidos en tres factores: depresión (ítems 3, 5, 10, 13, 16, 17 y 21), ansiedad (ítems 2, 4, 7, 9, 15, 19 y 20) y estrés (ítems 1, 6, 8, 11, 12, 14 y 18). Los ítems de la DASS-21 tienen una escala de respuesta Likert de cuatro puntos que van desde 0 (No se aplicó de ninguna manera) a 3 (Se aplicó mucho o la mayor parte del tiempo)^(39)^. Varios estudios demostraron las propiedades psicométricas de la DASS-21 en los profesionales de la salud^(41-42)^.

La satisfacción laboral se evaluó a través de la siguiente pregunta presente en el instrumento de caracterización de los participantes: En una escala de 1 a 10, indique qué tan satisfecho está con su trabajo en enfermería (1=muy insatisfecho y 10=muy satisfecho).

### Procedimientos y análisis estadístico

Siguiendo las recomendaciones de los *Standards for Educational and Psychological Testing* ^(43)^, las propiedades psicométricas del BAT – Versión general se evaluaron mediante el análisis de validez basado en la estructura interna, en las relaciones con medidas externas y en el proceso de respuesta a los ítems.

El análisis de validez basado en la estructura interna se realizó mediante la validez de constructo factorial, convergente y divergente. También se evaluaron la invarianza factorial y la confiabilidad de los datos. La validez factorial se evaluó mediante Análisis Factorial Confirmatorio (AFC), precedido por el análisis de sensibilidad psicométrico de los datos, considerando que los valores absolutos de asimetría (sk) y curtosis (ku) debían ser inferiores a 3 y 7, respectivamente, para garantizar el supuesto de la distribución normal de los datos requerido por el método del modelo de ecuaciones estructurales^(44)^. Para realizar el AFC se utilizó el *Weighted Least Squares Means and Variance* (WLSMV) y los siguientes índices para evaluar la calidad de ajuste de los modelos: *Comparative Fit Index* (CFI), *Tucker-Lewis Index* (TLI), *Root Mean Square Error of Approximation* (RMSEA) con un intervalo de confianza (IC) del 90% y *Standardized Root Mean Square Residual* (SRMR), considerado adecuado si CFI y TLI>0,90, RMSEA< 0,10 y SRMR<0,08^(45-46)^. También se evaluaron los pesos factoriales (λ) de los ítems y se consideraron adecuados si λ≥0,50. Se probaron seis modelos factoriales del BAT – Versión general en el AFC: BAT-C (cuatro factores de primer orden que corresponden a los síntomas primarios correlacionados, 22 ítems), BAT-S (dos factores de primer orden que corresponden a los síntomas secundarios correlacionados, 10 ítems), BAT32-6 (seis factores de primer orden que corresponden a todos síntomas primarios y secundarios correlacionados, 32 ítems), BAT32 MJSO (modelo jerárquico de segundo orden, compuesto por los seis factores de primer orden correlacionados y por un factor de segundo orden representado por el constructo *burnout*), BAT32-5 (cuatro factores de primer orden que corresponden a los síntomas primarios correlacionados y un factor compuesto por los 10 síntomas secundarios, 32 ítems) y BAT12 (versión corta compuesta por cuatro factores de primer orden que corresponden a los síntomas primarios correlacionados, 12 ítems)^(18)^.

La validez convergente del modelo factorial se estimó utilizando la varianza extraída de la media (VEM) de los factores del BAT – Versión general, se consideró adecuada una VEM≥0,50^(47)^. La validez discriminante del modelo factorial se analizó comparando la VEM de cada factor con el cuadrado de la correlación entre los factores, se considera que hay validez discriminante cuando la VEM de cada factor es mayor o igual al cuadrado de la correlación entre los factores (VEMi y VEMj≥ ρij^2^)^(47)^. La invarianza factorial del modelo se probó entre muestras independientes mediante el análisis multigrupo y la prueba de diferencia del CFI (ΔCFI). Para ello, la muestra se dividió aleatoriamente en dos subgrupos (prueba *n*=1799; validación *n*=1795) y se analizaron los valores del CFI de los modelos configural (M_0_), métrico (M_1_) y escalar (M_2_). Los valores de ΔCFI <0,01 (ΔCFI_M1-M0_ y ΔCFI_M2-M1_) se consideraron indicadores de invarianza factorial^(45,48)^. La invarianza factorial del BAT – Versión general también se evaluó según la presencia o ausencia de problemas emocionales/psicológicos de salud (presencia *n*=2500; ausencia *n*=1071) y según el sexo. Teniendo en cuenta la alta discrepancia relacionada con el número de participantes del sexo masculino y del sexo femenino, se seleccionó aleatoriamente una submuestra más pequeña (20%) de mujeres para realizar la comparación entre los grupos (masculino *n*=484; femenino *n*=622). La confiabilidad de los datos se evaluó mediante el coeficiente alfa ordinal (α) y la confiabilidad compuesta (CC), considerando que los valores de α y CC ≥0,70 eran indicadores de confiabilidad adecuada^(47)^.

La evidencia de validez basada en las relaciones con variables externas se evaluó mediante el análisis de la validez convergente de las correlaciones latentes que hay entre los factores del BAT – Versión general, las demandas y los recursos personales/laborales. La versión adaptada al contexto brasileño de la *Depression, Anxiety and Stress Scale* (DASS-21)^(39)^ se utilizó para estimar la correlación entre los síntomas de *burnout* y de depresión, ansiedad y estrés y presentó ajuste adecuado para la muestra [λ=0,64-0, TLI=0,968, CFI=0,971, RMSEA=0,078 (IC90%=0,076-0,080), SRMR=0,036 y α=0,92-0,94]. Para evaluar la correlación entre los síntomas de *burnout* y la satisfacción general con la vida, se utilizó la versión brasileña de la *Satisfaction with Life Scale* (SWLS)^(35)^, que también presentó un adecuado ajuste para la muestra [λ=0,69-0,89, TLI=0,990, CFI=0,995, RMSEA=0,092 (IC90%=0,080-0,104), SRMR=0,017 y α=0,89].

La validez basada en el proceso de respuesta a los ítems se evaluó mediante el análisis del funcionamiento diferencial de los ítems (*Differential Item Functioning* – DIF) entre dos grupos muestrales diferentes: presencia (*n*=2500) de problemas emocionales/psicológicos de salud y ausencia (*n*=1071) de problemas emocionales/psicológicos. La clasificación de estos grupos se basó en la respuesta a la siguiente pregunta: “¿En los últimos 30 días has trabajado con algún problema emocional/psicológico?”, presente en el instrumento de caracterización de la muestra y cuya respuesta era dicotómica (sí y no).

El DIF se estimó mediante análisis de regresión logística ordinal, en base a la estadística chi-cuadrado de la razón de verosimilitud (nivel de significación del 1%) y utilizando el *Partial-Credit Model* (PCM). También se estimaron los índices *Information-Weighted Mean Square* (Infit) y *Unweighted Mean Square* (Outfit) del modelo factorial completo del BAT – Versión general (BAT32-6). Los valores de Infit y Outfit de los ítems entre 0,5 y 1,5 se consideraron indicativos de ajuste al PCM^(49-50)^. Para evaluar el tamaño del efecto del análisis de DIF se utilizaron los pseudo coeficientes R^2^ de McFadden y Negelkerke, considerados insignificantes si R^2^<0,13^(51)^; los ítems que presentaron un efecto total significativo (*p*<0,01) se consideraron no equivalentes.

Para los análisis estadísticos se utilizaron los programas *IBM SPSS Statistics* 22 (IBM Corp., Armonk, N.Y., EE. UU.) y R^(50)^ con los paquetes *lavaan* (versión 0.6-10) ^(52)^, *SemTools* (versión 0.5-5)^(53)^ y *lordif* ^(51)^.

## Resultados

La mayoría de los participantes eran del sexo femenino (*n*=3090; 85,98%); 1789 (49,78%) estaban casados; 3006 (83,64%) tenían edades entre 19 y 44 años (promedio de edad = 35,75; DE = 10,09); 1722 (47,91%) eran enfermeros y 1862 (51,81%) eran técnicos y auxiliares de enfermería; 2468 (68,67%) tenían de 1 a 10 años de experiencia profesional; 1823 (50,72%) trabajaban en servicios hospitalarios y 847 (23,57%) en unidades de atención primaria de salud.

En el análisis de validez basado en la estructura interna, la sensibilidad psicométrica obtuvo valores adecuados de asimetría (sk=-0,49-1,42) y curtosis (ku=-1,30-1,81), lo que comprueba la distribución normal de los datos. El Análisis Factorial Confirmatorio (AFC) y el análisis de confiabilidad de los seis modelos factoriales alternativos del BAT – Versión general se presentan en la Tabla 1.

**Tabla 1:** - Análisis Factorial Confirmatorio y confiabilidad de los modelos alternativos del BAT – Versión general (n* *= 3594). Brasil, 2024

**Modelo**	λ*	**TLI** ^†^	**CFI** ^‡^	**RMSEA** ^§^ **[IC90%** ^||^ **]**	**SRMR** ^¶^	VEM**	**CC** ^††^	**α** ^‡‡^
BAT-C^§§^	0,768-0,927	0,964	0,969	0,092 [0,090-0,094]	0,040	0,732-0,769	0,916-0,964	0,910-0,961
BAT-S^||||^	0,719-0,920	0,972	0,979	0,099 [0,094-0,104]	0,039	0,608-0,661	0,886-0,906	0,881-0,897
BAT32-6^¶¶^	0,711-0,940	0,961	0,965	0,073 [0,071-0,074]	0,035	0,609-0,769	0,886-0,964	0,881-0,961
BAT32-5^***^	0,706-0,937	0,957	0,961	0,077 [0,075-0,078]	0,039	0,590-0,769	0,916-0,964	0,910-0,961
BAT12^†††^	0,790-0,924	0,981	0,986	0,084 [0,080-0,088]	0,032	0,704-0,824	0,877-0,934	0,872-0,931
BAT32 MJSO^‡‡‡^	0,710-0,941	0,955	0,959	0,078 [0,077-0,079]	0,044	0,609-0,769	0,886-0,964	0,881-0,961

*λ = Peso factorial; ^†^TLI = *Tucker-Lewis index*; ^‡^CFI = *Comparative Fit index*; ^§^RMSEA = *Root Mean Square Error of Approximation*; ^||^IC90% = 90% Intervalo de Confianza; ^¶^SRMR = *Standardized Root Mean Square Residual*; **VEM = Varianza extraída de la media; ^††^CC = Confiabilidad compuesta; ^‡‡^α = Coeficiente alfa ordinal; ^§§^BAT-C = Síntomas primarios; ^||||^BAT-S = Síntomas secundarios; ^¶¶^BAT32-6 = Síntomas primarios y secundarios; ^***^BAT32-5 = Síntomas primarios y 1 fator con 10 síntomas secundarios; ^†††^BAT12 = Versión corta de los síntomas primarios; ^‡‡‡^BAT32 MJSO = Modelo jerárquico de segundo orden

Los resultados confirmaron el ajuste adecuado de todos los modelos probados. Sin embargo, el modelo factorial completo del instrumento (BAT32-6) presentó el mejor ajuste a los datos [λ=0,711-0,940; TLI=0,961; CFI=0,965; RMSEA=0,073 (IC90%=0,071-0,074); SRMR=0,035] y fue elegido para continuar con los análisis.

Se confirmó la validez convergente de los factores del BAT32-6 (VEM=0,609-0,769); pero no se puedo confirmar la validez discriminante entre los factores *distress* psicológico y agotamiento (r^2^=0,716, *p*<0,001); *distress* psicológico y deterioro cognitivo (r^2^=0,719, *p*<0,001); *distress* psicológico y quejas psicosomáticas (r^2^=0,740, *p*<0,001); quejas psicosomáticas y agotamiento (r^2^=0,616, *p*<0,001), debido a las fuertes correlaciones entre ellos. La confiabilidad del BAT32-6 también fue adecuada para la muestra.

En lo que respecta a la evidencia de validez basada en las relaciones con medidas externas, la Tabla 2 presenta las correlaciones entre los dominios del BAT32-6 y las variables satisfacción general con la vida y satisfacción laboral. Se confirmó que el *burnout* y todos los factores del BAT presentaron correlaciones positivas con los síntomas de depresión, ansiedad y estrés (demandas) y negativas con satisfacción general con la vida y con la satisfacción laboral (recursos), como se esperaba, lo que confirma los supuestos teóricos del modelo JD-R.

**Tabla 2:** - Correlaciones entre dominios del BAT32-6 y variables externas (n = 3594). Brasil, 2024

	AG*	**DM** ^†^	**DM** ^‡^	**DC** ^§^	**DP** ^||^	**QP** ^¶^	***BURNOUT*** **
**DEPR** ^††^	0,779	0,751	0,584	0,771	0,774	0,671	0,849
**ANS** ^‡‡^	0,711	0,636	0,534	0,723	0,782	0,789	0,812
**STRES** ^§§^	0,779	0,676	0,606	0,812	0,827	0,706	0,865
**SWLS** ^||||^	-0,472	-0,455	-0,347	-0,432	-0,459	-0,418	-0,505
**SATLAB** ^¶¶^	-0,492	-0,623	-0,376	-0,421	-0,442	-0,395	-0,529

*AG = Agotamiento; ^†^DM = Distancia mental; ^‡^DM = Deterioro mental; ^§^DC = Deterioro cognitivo; ^||^DP = *Distress* psicológico; ^¶^QP = Quejas psicosomáticas; **BURNOUT = Modelo jerárquico de segundo orden; ^††^DEPR = Depresión; ^‡‡^ANS = Ansiedad; ^§§^STRES = Estrés; ^||||^SWLS = Satisfacción general con la vida; ^¶¶^SATLAB = Satisfacción laboral. Todas las correlaciones presentaron *p*<0,01

En la Tabla 3 se presenta el análisis de invarianza del BAT32-6 en muestras independientes (prueba y validación) y en función de la aparición de problemas emocionales/psicológicos de salud (presencia y ausencia). Se observó una invarianza de medición fuerte/estricta entre los modelos configural, métrico y escalar de las diferentes muestras, lo que refuerza la consistencia interna del instrumento.

**Tabla 3:** - Análisis de invarianza del BAT32-6 en diferentes muestras (n = 3594). Brasil, 2024

**Muestras**	**n**	λ*	**TLI** ^†^	**CFI** ^‡^	**RMSEA** ^§^ **[IC90%** ^||^ **]**	**SRMR** ^¶^	α**
Prueba	1799	0,714-0,945	0,964	0,967	0,070 [0,068-0,072]	0,036	0,879-0,961
Validación	1795	0,708-0,935	0,962	0,965	0,072 [0,070-0,074]	0,037	0,883-0,961
Invarianza (CFI)^¶^M_0_: Configural=0,966; M_1_: Métrico=0,967; M_2_: Escalar=0,968; ∆CFI<0,01 (M_1_-M_0_; M_2_-M_1_)							
Masculino	484	0,763-0,948	0,967	0,970	0,070 [0,066-0,074]	0,041	0,913-0,963
Femenino	622	0,727-0,945	0,967	0,970	0,065 [0,062-0,069]	0,040	0,877-0,962
Invarianza (CFI) ^¶^M_0_: Configural=0,970; M_1_: Métrico=0,970; M_2_: Escalar=0,969; ∆CFI<0,01 (M_1_-M_0_; M_2_-M_1_)							
+ Problemas emocionales^††^	2500	0,654-0,926	0,950	0,955	0,072 [0,071-0,074]	0,040	0,854-0,950
- Problemas emocionales^‡‡^	1071	0,685-0,929	0,959	0,963	0,065 [0,063-0,068]	0,043	0,867-0,956
Invarianza (CFI) ^¶^M_0_: Configural=0,957; M_1_: Métrico=0,957; M_2_: Escalar=0,960; ∆CFI<0,01 (M_1_-M_0_; M_2_-M_1_)							

*λ = Peso factorial; ^†^TLI = *Tucke*r*-Lewis index*; ^‡^CFI = *Comparative Fit index*; ^§^RMSEA = *Root Mean Square Error of Approximation*; ^||^IC90% = 90% Intervalo de Confianza; ^¶^SRMR = *Standardized Root Mean Square Residual*; **α = Coeficiente alfa ordinal; ^††^+ Problemas emocionales = Presencia de problemas emocionales/psicológicos de salud; ^‡‡^- Problemas emocionales = Ausencia de problemas emocionales/psicológicos de salud

También es importante destacar el AFC del BAT32 MJSO, que demostró que todos los factores reflejaban significativamente el *burnout* (β=0,769-0,942, *p*<0,001), especialmente el *distress* psicológico (β=0,942, *p*<0,001), el agotamiento (β =0,888, *p*<0,001) y el deterioro cognitivo (β=0,891, *p*<0,001).

Con respecto a la evidencia de validez basada en el proceso de respuesta a los ítems, la Tabla 4 presenta los índices de ajuste del BAT (valores de *Infit* y *Outfit*) y los resultados del análisis del DIF entre los subgrupos de la muestra (presencia o ausencia de problemas emocionales/psicológicos de salud).

**Tabla 4:** - Índices de ajuste y análisis del funcionamiento diferencial de los ítems entre subgrupos de la muestra (n = 3594). Brasil, 2024

**Ítem**	Subgrupo 1*		**Subgrupo 2** ^†^		**DIF**	**Pseudo R** ^2^	
	***Infit***	***Outfit***	***Infit***	***Outfit***	***p***-**valor**	**McFadden**	**Nagelkerke**
1	0,803	0,800	0,820	0,819	0,000	0,002	0,002
2	0,942	0,946	1,065	1,173	0,079	0,001	0,001
3	0,7881	0,788	0,777	0,779	0,039	0,001	0,001
4	0,815	0,793	0,846	0,848	0,370	0,000	0,000
5	0,792	0,777	0,797	0,770	0,625	0,000	0,000
6	0,775	0,760	0,702	0,655	0,917	0,000	0,000
7	0,830	0,816	0,849	0,842	0,004	0,001	0,001
8	0,771	0,753	0,776	0,767	0,329	0,000	0,000
9	0,909	0,910	1,031	1,058	0,199	0,000	0,000
10	1,090	1,229	1,252	2,308	0,199	0,000	0,000
11	1,031	1,071	1,053	1,031	0,953	0,000	0,000
12	1,171	1,308	1,205	1,675	0,103	0,000	0,001
13	0,942	0,927	0,938	0,895	0,058	0,001	0,001
14	1,075	1,117	1,183	1,391	0,013	0,001	0,001
15	1,110	1,122	1,105	1,150	0,000	0,003	0,005
16	0,985	0,974	1,026	1,018	0,000	0,002	0,003
17	1,137	1,079	1,062	1,093	0,003	0,001	0,003
18	0,841	0,820	0,840	0,812	0,838	0,000	0,000
19	0,873	0,853	0,746	0,659	0,322	0,000	0,000
20	1,097	1,112	1,027	1,055	0,202	0,000	0,001
21	0,764	0,768	0,731	0,698	0,003	0,001	0,001
22	0,895	0,914	0,852	0,827	0,892	0,000	0,000
23	1,276	1,369	1,233	1,634	0,068	0,001	0,001
24	0,904	0,929	0,937	0,959	0,175	0,000	0,000
25	0,692	0,691	0,680	0,683	0,000	0,002	0,001
26	1,000	1,007	0,944	0,936	0,000	0,003	0,005
27	1,150	1,273	1,227	1,382	0,105	0,000	0,001
28	1,172	1,210	1,138	1,227	0,001	0,002	0,003
29	1,263	1,372	1,185	1,249	0,359	0,000	0,000
30	1,243	1,324	1,209	1,233	0,118	0,000	0,001
31	1,163	1,214	1,198	1,225	0,186	0,000	0,001
32	1,099	1,118	1,018	0,954	0,139	0,000	0,001

*Subgrupo 1 = Presencia de problemas emocionales/psicológicos de salud; ^†^Subgrupo 2 = Ausencia de problemas emocionales/psicológicos de salud

Los resultados del análisis del DIF indicaron que, en el subgrupo 1 (presencia de problemas emocionales/psicológicos de salud), todos los ítems presentaron ajuste adecuado al PCM, (valores de *Infit* y *Outfit* entre 0,5 y 1,5). En el subgrupo 2 (ausencia de problemas emocionales/psicológicos de salud), los ítems 10, 12 y 23 presentan valores de *Outfit* > 1,5 (no adecuación al PCM). Además, los ítems 1, 7, 14, 15, 16, 17, 21, 25, 26 y 28 se consideraron no equivalentes, lo que indica que fueron respondidos de manera diferente por los participantes de cada subgrupo (*p*<0,01). Sin embargo, el tamaño del efecto del DIF en ambos subgrupos se consideró insignificante para todos los ítems (R^2^<0,13).

La Figura 1 muestra la distribución del rasgo latente de los participantes y la Curva Característica de los Ítems (CCI) en ambos subgrupos (presencia y ausencia de problemas emocionales/psicológicos de salud).

**Figura 1:** - (a) distribución del rasgo latente de los participantes; (b) Curvas Características de los Ítems **-** para todos los ítems y solo para los ítems que presentaron DIF; (c) *boxplot* de la distribución del rasgo latente en ambos subgrupos (n = 3594). Brasil, 2024

La distribución del rasgo latente en cada subgrupo (Figuras 1a y 1c) mostró un nivel mayor de *theta* (θ) entre los participantes que afirmaron tener problemas emocionales/psicológicos (subgrupo 1), indicando que el instrumento parece discriminar adecuadamente a los individuos con y sin problemas de salud emocional. Las CCI (Figura 1b) se mostraron coincidentes cuando los ítems son analizados en conjunto, confirmando la invariancia del modelo entre los subgrupos.

## Discusión

Este estudio confirmó las excelentes propiedades psicométricas del BAT – Versión general aplicada a una muestra de trabajadores de enfermería brasileños.

En el análisis de validez basado en la estructura interna, los resultados del AFC confirmó el adecuado ajuste de todos los modelos factoriales del BAT – Versión general en la muestra: modelo completo, compuesto por 32 ítems y seis factores correlacionados (BAT32-6); modelo compuesto por 23 ítems y los cuatro síntomas primarios (BAT-C); modelo jerárquico de segundo orden (BAT32 MJSO); y versión corta, compuesta por 12 ítems (BAT12). Sin embargo, el BAT32-6 representó la alternativa más sólida para evaluar los síntomas de *burnout* en los trabajadores de enfermería brasileños.

Esos resultados coinciden con la evidencia científica relacionada con la validación del BAT – Relacionado con el trabajo, que confirmaron las propiedades psicométricas de los modelos de primer y segundo orden compuestos por los factores primarios (BAT-C)^(24,28,54-55)^ y de la versión corta BAT12^(21,25)^ y destacan que se puede utilizar la versión compuesta por 12 ítems para evaluar los síntomas de *burnout* en diferentes contextos laborales y clínicos. Elegir entre utilizar la versión completa o la versión corta del instrumento dependerá del contexto laboral que será evaluado y del objetivo propuesto (detección rápida, seguimiento o monitoreo de los síntomas de *burnout* de los trabajadores).

Las correlaciones entre los factores del BAT32-6 también confirmaron la validez de constructo convergente y reforzaron los supuestos teóricos del instrumento, que considera el *burnout* como un síndrome caracterizado por cuatro dimensiones de síntomas primarios y dos dimensiones de síntomas secundarios interrelacionadas^(18)^.

En cuanto a la validez basada en medidas externas, los resultados demostraron que había correlaciones positivas entre los síntomas de *burnout* y los síntomas de depresión, ansiedad y estrés (demandas) y correlaciones negativas entre los síntomas de *burnout* y los constructos satisfacción general con la vida y satisfacción laboral (recursos), lo que confirma los supuestos del modelo *Job Demands–Resources* (JD-R) como marco conceptual. Según el modelo JD-R, las demandas representan factores relacionados con el trabajo y aspectos personales que generan estrés laboral y, a largo plazo, pueden provocar el desarrollo de *burnout*, los recursos personales y laborales se consideran factores protectores de la salud que desempeñan un papel mediador de extrema importancia y consisten en factores físicos, psicológicos, sociales u organizacionales que pueden (a) reducir el desgaste físico y psicológico asociado a las demandas laborales, (b) ayudar a los trabajadores a alcanzar las metas laborales y (c) estimular el crecimiento y desarrollo personal^(13,56)^. En este sentido, los recursos pueden reducir el impacto de las demandas y son importantes predictores de motivación, compromiso, satisfacción y placer en el trabajo^(57)^.

Comprender de forma teórica el proceso salud-enfermedad relacionado con el trabajo desde la perspectiva de la interacción entre las demandas y los recursos individuales y laborales representa el motivo principal para darle un mayor uso y para mejorar el modelo JD-R^(15)^. Con el paso de los años, aspectos intrínsecos al sujeto, como la satisfacción con la vida^(17)^, el compromiso con en el trabajo^(56)^ e incluso la resiliencia^(58)^, se fueron incorporando al JD-R como formas de recursos^(16)^.

En este sentido, se destacan las correlaciones negativas que se encontraron entre la satisfacción general con la vida, medida por la *Satisfaction with Life Scale* (SWLS), los síntomas de *burnout*, y los síntomas de depresión, ansiedad y estrés en la muestra. La satisfacción con la vida se puede considerar como una percepción global de experiencia individual de vida^(59)^ y a menudo se asocia con el bienestar y se la considera un factor protector contra los síntomas psicológicos^(60)^. Estos resultados coinciden con los de un estudio de validación del BAT – Relacionado con el trabajo que discute teóricamente la relación negativa entre estos constructos y el papel mediador de la satisfacción general con la vida en lo que respecta al *burnout* ^(17)^. También se comprobó que hay correlaciones negativas entre los síntomas de depresión, ansiedad y estrés y la satisfacción con la vida en los enfermeros^(17,61)^.

Además, es importante destacar las características del contexto social de los participantes de esta investigación, es decir, trabajadores de enfermería que vivieron todas las dificultades relacionadas con la pandemia de covid-19 mientras trabajaban en la primera línea de atención y que todavía sufren las consecuencias de esa crisis sanitaria en Brasil. Esas condiciones sociales y laborales pueden haber afectado seriamente la satisfacción general con la vida de ese grupo de trabajadores.

Las correlaciones positivas encontradas entre los síntomas de *burnout* y de depresión, ansiedad, estrés y en este estudio coinciden con la evidencia de estudios realizados con profesionales de la salud en Brasil^(62)^ y en Estambul^(63)^. En Portugal^(64)^, un estudio realizado con profesionales de la salud confirmó que había correlaciones positivas entre depresión y *burnout* y negativas entre agotamiento depresión y resiliencia, lo que sugiere que la resiliencia tiene un papel mediador en la reducción de dichos síntomas. Desde la perspectiva del JD-R, la resiliencia representa un recurso individual que los profesionales de enfermería pueden utilizar^(58)^.

Sin embargo, en los estudios mencionados, el BAT no se utilizó para evaluar la relación entre los síntomas de *burnout,* depresión ansiedad y estrés; sino que se utilizaron otros instrumentos psicométricos, como el *Copenhagen Burnout Inventory* ^(63-64)^ y el *Maslach Burnout Inventory* ^(62-63)^. Este hecho revela una debilidad importante en la comparación de los resultados y, al mismo tiempo, reafirma la originalidad de este estudio, ya que, hasta la fecha, no se ha encontrado evidencia sobre el uso concomitante del BAT y de la DASS-21 en profesionales de la salud.

En lo que respeta a la correlación negativa entre los síntomas de *burnout* y la satisfacción laboral, los resultados confirman los hallazgos de estudios de validación del BAT – Relacionado con el trabajo^(21,24-25,28)^ y demuestran la relación entre la satisfacción laboral y la enfermedad psicológica en trabajadores de enfermería^(3)^. Asimismo, las evidencias científicas confirman que hay correlaciones negativas entre la satisfacción laboral y los síntomas de depresión, ansiedad y estrés^(65-66)^.

En cuanto a las evidencias de validez basadas en el proceso de respuesta a los ítems, los resultados revelaron que el BAT32-6 funcionó adecuadamente en ambos subgrupos de trabajadores de enfermería, los que informaron que tenían problemas emocionales/psicológicos de salud y los que lo negaron, ello indica que el BAT – Versión general se puede utilizar tanto en la población normativa como en individuos afectados por problemas psicológicos.

Por último, cabe destacar que la principal contribución científica de este manuscrito es la presentación de un nuevo instrumento válido y confiable para evaluar los síntomas de *burnout* en profesionales de enfermería brasileños, que cuenta con un marco teórico sólido y actualizado, que coincide con las nuevas transformaciones que ocurrieron en los lugares de trabajo a nivel mundial.

En lo que respecta a las limitaciones de esta investigación, se destaca la escasez de estudios de validación del BAT – Versión general, lo que impide que se realice un análisis profundo y se comparen los resultados. Por otro lado, este hecho enfatiza la originalidad y relevancia del presente estudio.

## Conclusión

El BAT – Versión general demostró ser un instrumento robusto y consistente para medir los síntomas de *burnout* en trabajadores de enfermería brasileños. Por lo tanto, el BAT – Versión general se puede convertir en una importante herramienta de gestión para los líderes de enfermería y otros gestores de los servicios de salud que buscan implementar estrategias para promover ambientes de trabajo saludables, que incluyan aspectos físicos, psicológicos y emocionales de la salud de los trabajadores.

Por último, cabe destacar que los autores originales afirman que no hay que utilizar el BAT para el diagnóstico clínico del *burnout*. Sino que el instrumento es una herramienta valiosa que se puede utilizar en la evaluación individual de síntomas de *burnout*.

# Translation: Propriedades psicométricas do ***Burnout Assessment Tool*** – Versão geral em trabalhadores de enfermagem ^*^

**DOI:** 10.1590/1518-8345.7367.4426

**Authors:** Lacir José Santin, Bianca Gonzalez Martins, Juliana Alvares Duarte Bonini Campos, Ana Claudia Souza Vazquez, Maria Helena Palucci Marziale, Isabel Amelia Costa Mendes, Neyson Pinheiro Freire, Wilmar B. Schaufeli, Hans De Witte, Fernanda Ludmilla Rossi Rocha

**Affiliations:** 1 Universidade de São Paulo, Escola de Enfermagem de Ribeirão Preto, PAHO/WHO Collaborating Centre for Nursing Research Development, Ribeirão Preto, SP, Brasil.; 2 Universidade Estadual Paulista, Faculdade de Ciências Farmacêuticas, Araraquara, SP, Brasil.; 3 Universidade Federal de Ciências da Saúde, Departamento de Psicologia, Porto Alegre, RS, Brasil.; 4 Conselho Federal de Enfermagem, Brasília, DF, Brasil.; 5 Katholieke Universiteit Leuven, Faculty of Psychology and Educational Sciences, Leuven, Bélgica.; 6 Bolsista da Coordenação de Aperfeiçoamento de Pessoal de Nível Superior (CAPES), Brasil.; 7 Bolsista do Conselho Nacional de Desenvolvimento Científico e Tecnológico (CNPq), Brasil.

**Keywords:** Saúde Ocupacional, Enfermagem, Burnout, Psicometria, Esgotamento Psicológico, Burnout Assessment Tool

## Abstract

**Objetivo:**

analisar as evidências de validade do *Burnout Assessment Tool* (BAT) – Versão geral em uma amostra de trabalhadores de enfermagem brasileiros.

**Método:**

estudo transversal com amostragem não probabilística, realizado entre 3594 trabalhadores de enfermagem brasileiros. A validade do instrumento foi testada por meio da análise baseada na estrutura interna, nas relações com variáveis externas e no processo de resposta aos itens.

**Resultados:**

os modelos fatoriais do BAT – Versão geral mostraram adequado ajustamento aos dados. Entretanto, o modelo fatorial completo permitiu uma melhor compreensão da síndrome de *burnout* na amostra. Foi atestado que os fatores do BAT – Versão geral apresentaram correlações positivas com sintomas de depressão, ansiedade e estresse e negativas com a satisfação no trabalho e a satisfação geral com a vida. A análise das evidências de validade baseadas no processo de resposta aos itens revelou que o BAT – Versão geral funcionou adequadamente tanto no grupo de trabalhadores de enfermagem que relataram ter problemas emocionais ou psicológicos de saúde quanto no grupo de trabalhadores que negaram estes problemas.

**Conclusão:**

os resultados forneceram evidências robustas de validade do BAT – Versão geral em trabalhadores de enfermagem brasileiros.

## Destaques

**(1)** O BAT – Versão geral demonstrou ser um instrumento robusto e consistente.

**(2)** O BAT – Versão geral pode se tornar uma importante ferramenta de gerenciamento para gestores e líderes em enfermagem.

**(3)** É uma ferramenta que pode ser utilizada na avaliação individual de sintomas de *burnout.*

**(4)** O BAT – Versão geral não deve ser usado para o diagnóstico clínico de *burnout*.

## Introdução

Atualmente, o *burnout* se destaca como um dos principais distúrbios psicológicos relacionados ao estresse ocupacional crônico. Recentemente, ele foi postulado pela Organização Mundial da Saúde como uma doença ocupacional e incluído na Classificação Internacional de Doenças, CID-11^(1)^. A enfermagem é reconhecida como uma profissão essencial para a assistência médica voltada para as necessidades individuais^(2)^, cujas condições e demandas de trabalho estão diretamente relacionadas ao adoecimento físico e mental dos trabalhadores, evidenciando a síndrome de *burnout* ^(3-5)^. Durante a pandemia da covid-19, a demanda se intensificou, assim como as condições precárias de trabalho enfrentadas pelos profissionais de enfermagem em nível global, o que contribuiu para o aumento da prevalência de *burnout* e outros problemas psicológicos entre esses trabalhadores^(6-8)^.

Apesar das muitas definições, é praticamente unânime a opinião de que a exaustão representa o componente central do *burnout* ^(1,9-12)^. A definição mais atual e abrangente de *burnout* foi proposta em 2020^(12)^ com base no modelo *Job Demands–Resources* (JD-R)^(13)^ e em outras suposições teóricas específicas^(14)^. De acordo com o modelo JD-R, o *burnout* resulta de um desequilíbrio entre as demandas do trabalho (aspectos que exigem esforço físico ou mental e que, a longo prazo, podem culminar na exaustão do trabalhador) e os recursos do trabalho (fatores motivacionais e estratégias que os trabalhadores utilizam para lidar com suas demandas de trabalho)^(15-16)^.

Com base nessas estruturas teóricas^(12)^, foi proposto um novo instrumento, o *Burnout Assessment Tool* (BAT), que considera o *burnout* como uma síndrome composta por quatro sintomas primários (exaustão, distanciamento mental, prejuízo cognitivo e prejuízo emocional), que podem ser acompanhados por dois sintomas secundários (distresse psicológico e queixas psicossomáticas)^(12,17)^. De acordo com os autores, a exaustão se refere à perda severa de energia que resulta em exaustão mental, manifestada por sintomas como falta de energia para começar um novo trabalho; sensação de esgotamento total após um dia inteiro de trabalho; sensação de cansaço rápido, mesmo após um esforço mínimo no trabalho; e incapacidade de relaxar após o trabalho. O distanciamento mental representa o distanciamento psicológico do trabalho e uma forte relutância ou aversão ao trabalho. As principais características do distanciamento mental são a indiferença, as atitudes cínicas e a falta de interesse ou entusiasmo pelo trabalho. O prejuízo cognitivo se refere a um inadequado desempenho cognitivo, problemas de memória e déficits de atenção e concentração. Alguns sintomas específicos são a dificuldade de pensar com clareza ou de aprender coisas novas no trabalho, esquecimento, distração, falta de foco no trabalho. O prejuízo emocional envolve reações emocionais intensas, irritabilidade, frustração e raiva no trabalho, sentir-se chateado ou triste sem saber por quê; incapacidade de controlar as emoções no trabalho; e reações exageradas. Os sintomas secundários relacionados ao distresse psicológico referem-se a problemas psicológicos, como distúrbios do sono, sensação de tensão, ansiedade ou preocupação e flutuações de peso. As queixas psicossomáticas referem-se a sintomas físicos que são exacerbados ou resultantes de problemas psicológicos. Exemplos de tais sintomas são palpitações, dor no peito, problemas gástricos e intestinais, dores musculares e cefaleia^(18)^.

Originalmente, o BAT foi desenvolvido em duas versões: BAT – Versão geral, com 32 itens livres de contexto, e BAT – Relacionado ao trabalho, com 33 itens^(18)^. Basicamente, a diferença entre as versões geral e relacionada ao trabalho é que, com exceção do domínio distanciamento mental, os itens do BAT – Versão geral se referem à vida cotidiana do indivíduo e não ao contexto de trabalho^(18)^. O BAT – Relacionado ao trabalho tem sido usado em nível global e já foi culturalmente adaptado e validado em diversos contextos culturais^(19-25)^. Até o momento, não foram encontrados estudos que analisaram as propriedades psicométricas do BAT – Versão geral entre trabalhadores de enfermagem brasileiros.

Estudos de validação do BAT – Relacionado ao trabalho em amostras que incluem trabalhadores de diferentes setores da economia analisaram a adequação de diferentes modelos fatoriais do instrumento: (1) os quatro sintomas primários e dois sintomas secundários do *burnout* são analisados separadamente: BAT-C, modelo composto pelos quatro fatores que representam os sintomas primários do *burnout*, correlacionados; BAT-S, modelo composto pelos dois fatores que representam os sintomas secundários, correlacionados; (2) os sintomas primários e secundários do *burnout* são analisados em conjunto: BAT32, modelo completo composto por seis fatores, sendo estes representados pelos quatro sintomas primários e pelos dois sintomas secundários do *burnout*, correlacionados; (3) modelo hierárquico de segunda ordem (MHSO) do BAT32 (seis fatores de primeira ordem e um construto de segunda ordem - *burnout*). Exemplos são os estudos realizados em países da Europa^(21,26-27)^, Ásia^(23)^ e América do Sul^(28)^, incluindo o Brasil^(17,24,29)^. O BAT – Relacionado ao trabalho também foi aplicado em uma amostra composta por membros da equipe de enfermagem da Polônia^(19)^ e entre profissionais de saúde italianos^(27,30)^, demonstrando excelentes propriedades psicométricas nesses contextos e populações.

Evidências também apontaram que os sintomas primários do BAT – Relacionado ao trabalho apresentaram correlações positivas com as demandas do trabalho e negativas com os recursos do trabalho, reforçando os pressupostos teóricos do modelo JD-R^(13,31-32)^. Entre as principais demandas de trabalho que apresentaram correlações positivas com os sintomas primários do BAT estão: sobrecarga de trabalho; intenção de abandono do emprego ou da profissão^(21,24-25)^; pressão do tempo; conflito de papéis^(21)^; *workaholism* (trabalho excessivo e compulsivo)^(23)^; afeto negativo^(19,24)^; altas exigências mentais, emocionais e físicas^(17,28)^; burocracia; mudanças negativas e conflitos interpessoais^(17)^.

Em relação aos recursos do trabalho, foram encontradas correlações negativas entre os sintomas primários do BAT e as seguintes variáveis: engajamento no trabalho^(19,21,28)^; satisfação no trabalho^(19,21)^; autonomia, clareza de papéis e apoio dos colegas^(21,24-25)^; vigor, dedicação e absorção/concentração mental^(23)^; autoeficácia organizacional e autoestima^(29)^; comprometimento organizacional afetivo, otimismo, autoeficácia social e autoeficácia na realização de tarefas^(21)^; clareza no desenvolvimento das atividades de trabalho, apoio da equipe, supervisão, percepção espiritual, trabalho em equipe e controle do trabalho^(17,28)^; percepção sobre a tomada de decisões e esperança disposicional^(28)^.

Diante da escassez de estudos, o objetivo desta investigação foi analisar as evidências de validade do BAT – Versão geral em uma amostra de trabalhadores de enfermagem brasileiros.

## Método

### Desenho do estudo e amostra

Estudo transversal, com amostragem não probabilística. A população foi composta por enfermeiros, técnicos e auxiliares de enfermagem brasileiros. Para calcular o tamanho mínimo amostral necessário para realizar as análises estatísticas, foi considerada a necessidade de cinco a dez respondentes por parâmetro a ser estimado do modelo^(33)^. Considerando os 85 parâmetros do BAT – Versão geral (32 itens, 32 erros, seis fatores latentes e 15 correlações entre os fatores), o tamanho mínimo da amostra foi calculado entre 425 e 850 participantes. Foram considerados os seguintes critérios de inclusão: ser enfermeiro, técnico ou auxiliar de enfermagem e ter um endereço eletrônico (*e-mail*) registrado no Conselho Federal de Enfermagem (COFEN); estar exercendo a profissão atualmente; e ter trabalhado na área de enfermagem por pelo menos um ano.

Os profissionais foram convidados a participar por meio de uma mensagem eletrônica enviada pelo COFEN, que continha um *link* para acessar os instrumentos de coleta de dados disponíveis na plataforma eletrônica *Research Electronic Data Capture* (REDCap). Dados mais recentes indicam um total de 2.727.473 registros de profissionais de enfermagem no Brasil, dos quais 671.091 são enfermeiros; 447.375 são técnicos; e 1.608.653 são auxiliares de enfermagem^(34)^. No entanto, esses dados não correspondem ao número total de trabalhadores de enfermagem brasileiros, o que se deve à possibilidade de registros profissionais duplicados. Atualmente, 779.337 endereços de e-mail estão registrados no sistema COFEN, o que corresponde ao número total de mensagens eletrônicas enviadas neste estudo. Ao todo, 5979 profissionais de enfermagem concordaram voluntariamente em participar (taxa de adesão=0,77%) e foram identificados 3594 instrumentos preenchidos completamente, os quais compuseram a amostra (taxa de resposta=66,11%). A coleta de dados foi realizada entre 1º de abril e 31 de julho de 2022.

### Medidas

O *burnout* foi avaliado por meio da versão brasileira do *Burnout Assessment Tool* (BAT) – Versão geral, instrumento que foi adaptado culturalmente e validado para o contexto brasileiro pelos próprios autores originais (disponível em https://burnoutassessmenttool.be/project_eng/). No BAT – Versão geral, os sintomas centrais compreendem 22 itens e avaliam quatro dimensões primárias (exaustão, distanciamento mental, prejuízo cognitivo e prejuízo emocional); os sintomas secundários contêm 10 itens e avaliam duas dimensões (distresse psicológico e queixas psicossomáticas). Os itens possuem escala de resposta do tipo Likert de cinco pontos (1=Nunca, 2=Raramente, 3=Algumas vezes, 4=Com frequência e 5=Sempre)^(12)^. O escore global do instrumento pode ser avaliado em quatro níveis de *burnout*: baixo (< 25 percentil); moderado (de 25 a 75 percentil); alto (de 75 a 95 percentil); e muito alto (> 95 percentil), com base na pontuação média das respostas dos participantes^(18)^.

A satisfação geral com a vida foi avaliada por meio da versão brasileira da *Satisfaction with Life Scale* (SWLS)^(35)^. A SWLS foi originalmente desenvolvida em inglês para avaliar a percepção geral do indivíduo em relação à satisfação com a vida^(36)^ e possui cinco itens, respondidos por meio de uma escala do tipo Likert de sete pontos que varia de 1 (discordo totalmente) a 7 (concordo totalmente). Evidências têm comprovado as excelentes propriedades psicométricas do SWLS em diferentes contextos culturais^(37-38)^.

Os sintomas de depressão, ansiedade e estresse foram avaliados por meio da *Depression, Anxiety and Stress Scale* (DASS-21)^(39)^, originalmente desenvolvida em 1995^(40)^ e que apresenta 21 itens distribuídos em três fatores: depressão (itens 3, 5, 10, 13, 16, 17 e 21), ansiedade (itens 2, 4, 7, 9, 15, 19 e 20) e estresse (itens 1, 6, 8, 11, 12, 14 e 18). Os itens da DASS-21 possuem escala de resposta do tipo Likert de quatro pontos que varia de 0 (Não se aplicou de maneira alguma) a 3 (Aplicou-se muito ou na maioria do tempo)^(39)^. Diversos estudos demonstraram as propriedades psicométricas da DASS-21 entre profissionais de saúde^(41-42)^.

A satisfação no trabalho foi analisada por meio da seguinte questão existente no instrumento de caracterização dos participantes: Em uma escala de 1 a 10, indique o quanto você está satisfeito com seu trabalho na enfermagem (1=muito insatisfeito e 10=muito satisfeito).

### Procedimentos e análise estatística

Seguindo as recomendações dos *Standards for Educational and Psychological Testing* ^(43)^, as propriedades psicométricas do BAT – Versão geral foram avaliadas por meio da análise da validade baseada na estrutura interna, nas relações com medidas externas e no processo de resposta aos itens.

A análise da validade baseada na estrutura interna foi conduzida por meio da validade de construto fatorial, convergente e divergente. A invariância fatorial e a confiabilidade dos dados também foram avaliadas. A validade fatorial foi testada por meio da Análise Fatorial Confirmatória (AFC), precedida pela análise de sensibilidade psicométrica dos dados, considerando os valores absolutos de assimetria (sk) e curtose (ku) menores que 3 e 7, respectivamente, para garantir o pressuposto da distribuição normal dos dados exigido pelo método da modelagem de equações estruturais^(44)^. Para realizar a AFC, foi utilizado o *Weighted Least Squares Means and Variance* (WLSMV) e os seguintes índices para avaliar a qualidade do ajustamento dos modelos: *Comparative Fit Index* (CFI); *Tucker–Lewis Index* (TLI); *Root Mean Square Error of Approximation* (RMSEA) com intervalo de confiança (IC) de 90% e *Standardized Root Mean Square Residual* (SRMR), considerado adequado se CFI e TLI>0,90, RMSEA<0,10 e SRMR<0,08^(45-46)^. Os pesos fatoriais (λ) dos itens também foram avaliados e considerados adequados quando λ ≥ 0,50. Seis modelos fatoriais do BAT – Versão geral foram testados na AFC: BAT-C (quatro fatores de primeira ordem que correspondem aos sintomas primários correlacionados, 22 itens), BAT-S (dois fatores de primeira ordem que correspondem aos sintomas secundários correlacionados, 10 itens), BAT32-6 (seis fatores de primeira ordem que correspondem a todos os sintomas primários e secundários correlacionados, 32 itens), BAT32 MHSO (modelo hierárquico de segunda ordem, composto pelos seis fatores de primeira ordem correlacionados e por um fator de segunda ordem representado pelo construto *burnout*), BAT32-5 (quatro fatores de primeira ordem que correspondem aos sintomas primários correlacionados e um fator composto pelos 10 sintomas secundários, 32 itens) e BAT12 (versão reduzida composta por quatro fatores de primeira ordem que correspondem aos sintomas primários correlacionados, 12 itens)^(18)^.

A validade convergente do modelo fatorial foi estimada por meio da variância extraída média (VEM) dos fatores do BAT – Versão geral, considerada adequada se VEM≥0,50^(47)^. A validade discriminante do modelo fatorial foi analisada comparando a VEM de cada fator com o quadrado da correlação entre os fatores, considerando que há validade discriminante quando a VEM de cada fator for maior ou igual ao quadrado da correlação entre os fatores (VEMi e VEMj≥ρij^2^)^(47)^. A invariância fatorial do modelo foi testada entre amostras independentes por meio da análise multigrupos e do teste de diferença do CFI (ΔCFI). Assim, a amostra foi dividida aleatoriamente em dois subgrupos (teste *n*=1799; validação *n*=1795) e os valores de CFI dos modelos configural (M_0_), métrico (M_1_) e escalar (M_2_) foram analisados. Os valores de ΔCFI <0,01 (ΔCFI_M1-M0_ e ΔCFI_M2-M1_) foram considerados como indicativos de invariância fatorial^(45,48)^. A invariância fatorial do BAT – Versão geral também foi avaliada de acordo com a presença ou ausência de problemas emocionais/psicológicos de saúde (presença *n*=2500; ausência *n*=1071) e de acordo com o sexo. Considerando a alta discrepância relacionada ao número de participantes do sexo masculino e do sexo feminino, uma subamostra menor (20%) de mulheres foi selecionada aleatoriamente para comparação entre os grupos (masculino *n*=484; feminino *n*=622). A confiabilidade dos dados foi avaliada usando o coeficiente alfa ordinal (α) e a confiabilidade composta (CC), considerando valores de α e CC de ≥0,70 como indicativos de confiabilidade adequada^(47)^.

A evidência de validade baseada nas relações com variáveis externas foi avaliada por meio da análise da validade convergente das correlações latentes existentes entre os fatores do BAT – Versão geral, as demandas e os recursos pessoais/ocupacionais. Esperava-se que o *burnout* e seus sintomas primários e secundários apresentassem correlações positivas com os construtos depressão, ansiedade e estresse (demandas) e correlações negativas com a satisfação geral com a vida e com a satisfação no trabalho (recursos). A versão adaptada para o contexto brasileiro da *Depression, Anxiety and Stress Scale* (DASS-21)^(39)^ foi utilizada para estimar a correlação entre os sintomas de *burnout* e de depressão, ansiedade e estresse e apresentou ajustamento adequado para a amostra [λ=0,64-0,90, TLI=0,968, CFI=0,971, RMSEA=0,078 (IC90%=0,076-0,080), SRMR=0,036 e α=0,92-0,94]. Para avaliar a correlação entre os sintomas de *burnout* e a satisfação geral com a vida, foi utilizada a versão brasileira da *Satisfaction with Life Scale* (SWLS)^(35)^, que também apresentou adequado ajustamento para a amostra [λ=0,69-0,89, TLI=0,990, CFI=0,995, RMSEA=0,092 (IC90%=0,080-0,104), SRMR=0,017 e α=0,89].

A validade baseada no processo de resposta aos itens foi avaliada por meio da análise do funcionamento diferencial dos itens (*Differential Item Functioning* – DIF) entre dois grupos amostrais distintos: presença (*n*=2500) de problemas emocionais/psicológicos de saúde e ausência (*n*=1071) de problemas emocionais/psicológicos. A classificação desses grupos ocorreu com base na resposta à seguinte pergunta: “Nos últimos 30 dias, você trabalhou com algum problema emocional/psicológico?”, presente no instrumento de caracterização da amostra e cuja resposta era dicotômica (sim e não).

A DIF foi estimada por meio de análise de regressão logística ordinal, com base na estatística qui-quadrado da razão de verossimilhança (nível de significância de 1%) e utilizando-se o *Partial-Credit Model* (PCM). Os índices *Information-Weighted Mean Square* (*Infit*) e o *Unweighted Mean Square* (*Outfit*) do modelo fatorial completo do BAT – Versão geral (BAT32-6) também foram estimados. Valores de *Infit* e *Outfit* dos itens entre 0,5 e 1,5 foram considerados indicativos de ajuste ao PCM^(49-50)^. Para avaliar o tamanho do efeito da análise de DIF, foram utilizados os coeficientes pseudo R^2^ de McFadden e de Negelkerke, considerados insignificantes se R^2^<0,13^(51)^; itens que apresentaram efeito total significativo (*p*<0,01) foram considerados não equivalentes.

Para as análises estatísticas, foram utilizados os programas *IBM SPSS Statistics* 22 (IBM Corp., Armonk, N.Y., EUA) e R^(50)^, com os pacotes *lavaan* (versão 0.6-10)^(52)^, *SemTools* (versão 0.5-5)^(53)^, *lordif* ^(51)^ e eRm^(49)^.

## Resultados

A maioria dos participantes era do sexo feminino (*n*=3090; 85,98%); 1789 (49,78%) eram casados; 3006 (83,64%) tinham entre 19 e 44 anos (média de idade = 35,75; DP = 10,09); 1722 (47,91%) eram enfermeiros e 1862 (51,81%) eram técnicos e auxiliares de enfermagem; 2468 (68,67%) tinham de um a 10 anos de experiência profissional; 1823 (50,72%) trabalhavam em serviços hospitalares e 847 (23,57%) atuavam em unidades de atenção primária à saúde.

Na análise de validade baseada na estrutura interna, a sensibilidade psicométrica mostrou valores adequados de assimetria (sk=-0,49-1,42) e curtose (ku=-1,30-1,81), comprovando a distribuição normal dos dados. A Análise Fatorial Confirmatória (AFC) e a análise de confiabilidade dos seis modelos fatoriais alternativos do BAT – Versão geral são apresentadas na Tabela 1.

Os resultados confirmaram o adequado ajustamento de todos os modelos testados. Entretanto, o modelo fatorial completo do instrumento (BAT32-6) apresentou o melhor ajustamento aos dados [λ=0,711-0,940; TLI=0,961; CFI=0,965; RMSEA=0,073 (IC90%=0,071-0,074); SRMR=0,035] e foi eleito para a continuidade das análises.

**Tabela 1:** - Análise Fatorial Confirmatória e confiabilidade dos modelos alternativos do BAT – Versão geral (n = 3594). Brasil, 2024

**Modelo**	λ*	**TLI** ^†^	**CFI** ^‡^	**RMSEA** ^§^ **[IC90%** ^||^ **]**	**SRMR** ^¶^	VEM**	**CC** ^††^	**α** ^‡‡^
BAT-C^§§^	0,768-0,927	0,964	0,969	0,092 [0,090-0,094]	0,040	0,732-0,769	0,916-0,964	0,910-0,961
BAT-S^||||^	0,719-0,920	0,972	0,979	0,099 [0,094-0,104]	0,039	0,608-0,661	0,886-0,906	0,881-0,897
BAT32-6^¶¶^	0,711-0,940	0,961	0,965	0,073 [0,071-0,074]	0,035	0,609-0,769	0,886-0,964	0,881-0,961
BAT32-5^***^	0,706-0,937	0,957	0,961	0,077 [0,075-0,078]	0,039	0,590-0,769	0,916-0,964	0,910-0,961
BAT12^†††^	0,790-0,924	0,981	0,986	0,084 [0,080-0,088]	0,032	0,704-0,824	0,877-0,934	0,872-0,931
BAT32 MHSO^‡‡‡^	0,710-0,941	0,955	0,959	0,078 [0,077-0,079]	0,044	0,609-0,769	0,886-0,964	0,881-0,961

*λ = Peso fatorial; ^†^TLI = *Tucker-Lewis index*; ^‡^CFI = *Comparative Fit index*; ^§^RMSEA = *Root Mean Square Error of Approximation*; ^||^IC90% = 90% Intervalo de Confiança; ^¶^SRMR = *Standardized Root Mean Square Residual*; **VEM = Variância extraída média; ^††^CC = Confiabilidade composta; ^‡‡^α = Coeficiente alfa ordinal; ^§§^BAT-C = Sintomas primários; ^||||^BAT-S = Sintomas secundários; ^¶¶^BAT32-6 = Sintomas primários e secundários; ^***^BAT32-5 = Sintomas primários e 1 fator com 10 sintomas secundários; ^†††^BAT12 = Versão reduzida dos sintomas primários; ^‡‡‡^BAT32 MHSO = Modelo hierárquico de segunda ordem

A validade convergente dos fatores do BAT32-6 foi confirmada (VEM=0,609-0,769); no entanto, não foi possível confirmar a validade discriminante entre os fatores distresse psicológico e exaustão (r^2^=0,716, *p*<0.001), distresse psicológico e prejuízo emocional (r^2^=0,719, *p*<0,001), distresse psicológico e queixas psicossomáticas (r^2^=0,740, *p*<0,001), queixas psicossomáticas e exaustão (r^2^=0,616, *p*<0,001), devido às fortes correlações entre eles. A confiabilidade dos fatores do BAT32-6 também se mostrou adequada para a amostra.

Com relação à evidência de validade baseada nas relações com medidas externas, a Tabela 2 apresenta as correlações entre os fatores do BAT32-6 e as variáveis satisfação geral com a vida e satisfação no trabalho. Foi confirmado que o *burnout* e todos os fatores do BAT32-6 apresentaram correlações positivas com os sintomas de depressão, ansiedade e estresse (demandas) e correlações negativas com a satisfação geral com a vida e com a satisfação no trabalho (recursos), conforme esperado, apoiando os pressupostos teóricos do modelo JD-R.

**Tabela 2:** - Correlações entre os domínios do BAT32-6 e variáveis externas (n = 3594). Brasil, 2024

	EX*	**DM** ^†^	**PC** ^‡^	**PE** ^§^	**DP** ^||^	**QP** ^¶^	BURNOUT**
**DEPR** ^††^	0,779	0,751	0,584	0,771	0,774	0,671	0,849
**ANS** ^‡‡^	0,711	0,636	0,534	0,723	0,782	0,789	0,812
**STRES** ^§§^	0,779	0,676	0,606	0,812	0,827	0,706	0,865
**SWLS** ^||||^	-0,472	-0,455	-0,347	-0,432	-0,459	-0,418	-0,505
**SATISFTRAB** ^¶¶^	-0,492	-0,623	-0,376	-0,421	-0,442	-0,395	-0,529

*EX = Exaustão; ^†^DM = Distanciamento mental; ^‡^PC = Prejuízo cognitivo; ^§^PE = Prejuízo emocional; ^||^DP = Distresse psicológico; ^¶^QP = Queixas psicossomáticas; **BURNOUT = Modelo hierárquico de segunda ordem; ^††^DEPR = Depressão; ^‡‡^ANS = Ansiedade; ^§§^STRES = Estresse; ^||||^SWLS = Satisfação geral com a vida; ^¶¶^SATISFTRAB = Satisfação no trabalho. Todas as correlações apresentaram *p*<0,01

A análise de invariância do BAT32-6 em subgrupos independentes (teste e validação) e de acordo com a ocorrência de problemas emocionais/psicológicos de saúde (presença e ausência) é apresentada na Tabela 3. Foi observada invariância de medida forte/estrita entre os modelos de configural, métrico e escalar dos diferentes subgrupos, reforçando a consistência interna do instrumento.

**Tabela 3:** - Análise de invariância do BAT32-6 em diferentes grupos (n = 3594). Brasil, 2024

**Amostras**	**n**	λ*	**TLI** ^†^	**CFI** ^‡^	**RMSEA** ^§^ **[IC90%** ^||^ **]**	**SRMR** ^¶^	α**
Teste	1799	0,714-0,945	0,964	0,967	0,070 [0,068-0,072]	0,036	0,879-0,961
Validação	1795	0,708-0,935	0,962	0,965	0,072 [0,070-0,074]	0,037	0,883-0,961
Invariância (CFI)^¶^ M_0_:Configural=0,966; M_1_: Métrico=0,967; M_2_: Escalar=0,968; ∆CFI<0,01 (M_1_-M_0_; M_2_-M_1_)							
Masculino	484	0,763-0,948	0,967	0,970	0,070 [0,066-0,074]	0,041	0,913-0,963
Feminino	622	0,727-0,945	0,967	0,970	0,065 [0,062-0,069]	0,040	0,877-0,962
Invariância (CFI)^¶^ M_0_:Configural=0,970; M_1_: Métrico=0,970; M_2_: Escalar=0,969; ∆CFI<0,01 (M_1_-M_0_; M_2_-M_1_)							
+ Problemas emocionais^††^	2500	0,654-0,926	0,950	0,955	0,072 [0,071-0,074]	0,040	0,854-0,950
- Problemas emocionais^‡‡^	1071	0,685-0,929	0,959	0,963	0,065 [0,063-0,068]	0,043	0,867-0,956
Invariância (CFI)^¶^ M_0_:Configural=0,957; M_1_: Métrico=0,957; M_2_: Escalar=0,960; ∆CFI<0,01 (M_1_-M_0_; M_2_-M_1_)							

*λ = Peso fatorial; ^†^TLI = *Tucker-Lewis index*; ^‡^CFI = *Comparative Fit index*; ^§^RMSEA = *Root Mean Square Error of Approximation*; ^||^IC90% = 90% Intervalo de Confiança; ^¶^SRMR = *Standardized Root Mean Square Residual*; **α = Coeficiente alfa ordinal; ^††^+ Problemas emocionais = Presença de problemas emocionais/psicológicos de saúde; ^‡‡^- Problemas emocionais = Ausência de problemas emocionais/psicológicos

Considera-se importante destacar a AFC do BAT32 MHSO, que comprovou que o *burnout* foi fortemente refletido por todos os fatores (β=0,769-0,942, *p*<0,001), especialmente pelo distresse psicológico (β=0,942, *p*<0,001), pela exaustão (β=0,888, *p*<0,001) e pelo prejuízo emocional (β=0,891, *p*<0,001).

Em relação às evidências de validade baseadas no processo de resposta aos itens, a Tabela 4 apresenta os índices de ajustamento do BAT32-6 (valores de *Infit* e *Outfit*) e os resultados da análise da DIF entre subgrupos da amostra (presença ou ausência de problemas emocionais/psicológicos de saúde).

**Tabela 4:** - Índices de ajustamento e análise de funcionamento diferencial dos itens entre subgrupos amostrais (n = 3594). Brasil, 2024

**Item**	Subgrupo 1*		**Subgrupo 2** ^†^		**DIF**	**Pseudo R** ^2^	
	***Infit***	***Outfit***	***Infit***	***Outfit***	*p-* **valor**	**McFadden**	**Nagelkerke**
1	0,803	0,800	0,820	0,819	0,000	0,002	0,002
2	0,942	0,946	1,065	1,173	0,079	0,001	0,001
3	0,788	0,788	0,777	0,779	0,039	0,001	0,001
4	0,815	0,793	0,846	0,848	0,370	0,000	0,000
5	0,792	0,777	0,797	0,770	0,625	0,000	0,000
6	0,775	0,760	0,702	0,655	0,917	0,000	0,000
7	0,830	0,816	0,849	0,842	0,004	0,001	0,001
8	0,771	0,753	0,776	0,767	0,329	0,000	0,000
9	0,909	0,910	1,031	1,058	0,199	0,000	0,000
10	1,090	1,229	1,252	2,308	0,199	0,000	0,000
11	1,031	1,071	1,053	1,031	0,953	0,000	0,000
12	1,171	1,308	1,205	1,675	0,103	0,000	0,001
13	0,942	0,927	0,938	0,895	0,058	0,001	0,001
14	1,075	1,117	1,183	1,391	0,013	0,001	0,001
15	1,110	1,122	1,105	1,150	0,000	0,003	0,005
16	0,985	0,974	1,026	1,018	0,000	0,002	0,003
17	1,137	1,079	1,062	1,093	0,003	0,001	0,003
18	0,841	0,820	0,840	0,812	0,838	0,000	0,000
19	0,873	0,853	0,746	0,659	0,322	0,000	0,000
20	1,097	1,112	1,027	1,055	0,202	0,000	0,001
21	0,764	0,768	0,731	0,698	0,003	0,001	0,001
22	0,895	0,914	0,852	0,827	0,892	0,000	0,000
23	1,276	1,369	1,233	1,634	0,068	0,001	0,001
24	0,904	0,929	0,937	0,959	0,175	0,000	0,000
25	0,692	0,691	0,680	0,683	0,000	0,002	0,001
26	1,000	1,007	0,944	0,936	0,000	0,003	0,005
27	1,150	1,273	1,227	1,382	0,105	0,000	0,001
28	1,172	1,210	1,138	1,227	0,001	0,002	0,003
29	1,263	1,372	1,185	1,249	0,359	0,000	0,000
30	1,243	1,324	1,209	1,233	0,118	0,000	0,001
31	1,163	1,214	1,198	1,225	0,186	0,000	0,001
32	1,099	1,118	1,018	0,954	0,139	0,000	0,001

*Subgrupo 1 = Presença de problemas emocionais/psicológicos de saúde; ^†^Subgrupo 2 = Ausência de problemas emocionais/psicológicos

Os resultados da análise da DIF indicaram que, no subgrupo 1 (presença de problemas emocionais/psicológicos de saúde), todos os itens apresentaram ajuste adequado ao PCM (valores de *Infit* e *Outfit* entre 0,5 e 1,5). No subgrupo 2 (ausência de problemas emocionais/psicológicos de saúde), os itens 10, 12 e 23 apresentaram valores de *Outfit* > 1,5 (não adequação ao PCM). Além disso, os itens 1, 7, 14, 15, 16, 17, 21, 25, 26 e 28 foram considerados não equivalentes (*p*<0,01), indicando que foram respondidos de forma diferente pelos participantes de cada subgrupo. Entretanto, o tamanho do efeito da DIF em ambos os subgrupos foi considerado negligenciável para todos os itens (R^2^<0,13).

A Figura 1 mostra a distribuição do traço latente dos participantes e a Curva Característica dos Itens (CCI) em ambos os subgrupos (presença e ausência de problemas emocionais/psicológicos de saúde).

**Figura 1:** - (a) distribuição do traço latente dos participantes; (b) Curvas Características dos Itens – para todos os itens e somente para os itens que apresentaram DIF; (c) *boxplot* da distribuição do traço latente em ambos os subgrupos (n = 3594). Brasil, 2024

A distribuição do traço latente em cada subgrupo (Figuras 1a e 1c) mostrou um nível maior de *theta* (θ) entre os participantes que afirmaram possuir problemas emocionais/psicológicos (subgrupo 1), indicando que o instrumento parece discriminar adequadamente indivíduos com e sem problemas de saúde emocional. As CCI (Figura 1b) se mostraram coincidentes quando os itens são analisados em conjunto, confirmando a invariância do modelo entre os subgrupos.

## Discussão

Este estudo confirmou as excelentes propriedades psicométricas do BAT – Versão geral aplicado em uma amostra de trabalhadores de enfermagem brasileiros.

Na análise de validade baseada na estrutura interna, os resultados da AFC comprovaram o adequado ajustamento de todos os modelos fatoriais do BAT – Versão geral na amostra: modelo completo, composto por 32 itens e seis fatores correlacionados (BAT32-6); modelo composto por 23 itens e pelos quatro sintomas primários (BAT-C); modelo hierárquico de segunda ordem (BAT32 MHSO); e versão reduzida, composta por 12 itens (BAT12). Entretanto, o BAT32-6 representou a alternativa mais robusta para avaliar sintomas de *burnout* entre os trabalhadores de enfermagem brasileiros.

Esses resultados corroboram evidências científicas relacionadas à validação do BAT – Relacionado ao trabalho, as quais confirmaram as propriedades psicométricas dos modelos de primeira e de segunda ordem compostos pelos fatores primários (BAT-C)^(24,28,54-55)^ e da versão reduzida (BAT12)^(21,25)^, destacando a possibilidade de uso da versão composta por 12 itens para avaliar sintomas de *burnout* em diferentes contextos ocupacionais e clínicos. A escolha entre utilizar a versão completa ou a versão reduzida do instrumento dependerá do contexto laboral a ser estudado e do objetivo proposto (triagem rápida, monitoramento ou acompanhamento dos sintomas de *burnout* dos trabalhadores).

As correlações entre os fatores do BAT32-6 atestaram ainda a validade de construto convergente e reforçaram os pressupostos teóricos do instrumento, que consideram o *burnout* como uma síndrome caracterizada por quatro dimensões de sintomas primários e duas dimensões de sintomas secundários inter-relacionadas^(18)^.

Em relação à validade baseada em medidas externas, os resultados mostraram correlações positivas entre os sintomas de *burnout* e os sintomas de depressão, ansiedade e estresse (demandas) e correlações negativas entre os sintomas de *burnout* e os construtos satisfação geral com a vida e satisfação no trabalho (recursos), confirmando as suposições do modelo *Job Demands–Resources* (JD-R) como estrutura conceitual. De acordo com o JD-R, as demandas representam aspectos individuais, sociais e organizacionais relacionados ao trabalho que exigem esforço físico ou mental constante (estressores) e podem levar, a longo prazo, ao desenvolvimento da síndrome de *burnout*; os recursos representam aspectos físicos, psicológicos, sociais e organizacionais relacionados ao trabalho capazes de: (a) reduzir o desgaste físico e psicológico associado às demandas de trabalho; (b) auxiliar os trabalhadores a atingir as metas de trabalho; (c) estimular o crescimento e o desenvolvimento pessoais^(13,56)^. Nesse sentido, os recursos são entendidos como fatores de proteção à saúde que desempenham um papel mediador de extrema importância para mitigar os impactos das demandas de trabalho e predizer a motivação, o engajamento e a satisfação no trabalho^(13,57)^.

A compreensão teórica do processo saúde-doença relacionado ao trabalho a partir da perspectiva da interação entre as demandas e os recursos individuais e ocupacionais representa a principal razão para o aumento do uso e do aprimoramento do modelo JD-R^(15)^. Ao longo dos anos, aspectos intrínsecos ao próprio sujeito, como satisfação com a vida^(17)^, engajamento no trabalho^(56)^ e resiliência^(58)^ foram sendo reconhecidas como formas de recursos.

Neste sentido, ressaltam-se as correlações negativas encontradas entre a satisfação geral com a vida (medida pela *Satisfaction with Life Scale*) e os sintomas de *burnout*, depressão, ansiedade e estresse na amostra. A satisfação com a vida pode ser entendida como uma percepção global da experiência individual de vida^(59)^, frequentemente associada ao bem-estar e reconhecida como um fator de proteção contra sintomas psicológicos^(60)^. Esses resultados corroboram estudo de validação do BAT – Relacionado ao trabalho que discute teoricamente a relação entre esses construtos e o papel mediador da satisfação geral com a vida em relação ao *burnout* ^(17)^. Entre enfermeiros, as correlações negativas entre os sintomas de depressão, ansiedade e estresse e a satisfação geral com a vida também foram comprovadas^(17,61)^.

Em relação a isso, considera-se importante destacar as características do contexto social e dos participantes deste estudo, ou seja, trabalhadores de enfermagem que vivenciaram todas as dificuldades relacionadas ao enfrentamento da pandemia da covid-19 atuando na linha de frente do cuidado e que ainda sofrem as consequências dessa crise sanitária no Brasil. Essas condições sociais e ocupacionais podem ter afetado seriamente a satisfação geral com a vida destes profissionais.

As correlações positivas encontradas entre os sintomas de *burnout*, depressão, ansiedade e estresse corroboram evidências de estudos realizados entre trabalhadores de saúde no Brasil^(62)^, em Istambul^(63)^ e em Portugal^(64)^. Nesta última investigação, foram atestadas correlações positivas entre depressão e *burnout* e negativas entre exaustão, depressão e resiliência, sugerindo o papel mediador da resiliência na ocorrência desses sintomas. Na perspectiva do JD-R, a resiliência representa um recurso individual que pode ser utilizado pelos trabalhadores de enfermagem^(58)^.

Entretanto, nos estudos supracitados, o BAT não foi utilizado para avaliar a relação entre os sintomas, sendo utilizados outros instrumentos psicométricos, como o *Copenhagen Burnout Inventory* ^(63-64)^ e o *Maslach Burnout Inventory* ^(62-63)^. Esse fato revela uma importante fragilidade na comparação dos resultados e, ao mesmo tempo, reafirma a originalidade deste estudo, uma vez que, até o momento, não foram encontradas evidências sobre o uso concomitante do BAT e da DASS-21 entre profissionais de saúde.

Sobre as correlações negativas entre as variáveis latentes investigadas, os resultados corroboram os achados de estudos de validação do BAT – Relacionado ao trabalho^(21,24-25,28)^ relacionados aos sintomas de *burnout* e à satisfação no trabalho, bem como as evidências sobre a relação entre a satisfação no trabalho e os sintomas de depressão, ansiedade e estresse^(65-66)^.

Em relação às evidências de validade baseadas no processo de resposta aos itens, os resultados mostraram que o BAT32-6 funcionou adequadamente em ambos os subgrupos de trabalhadores de enfermagem, aqueles que relataram e aqueles que negaram problemas emocionais/psicológicos de saúde, indicando que o BAT – Versão geral pode ser utilizado tanto na população normativa quanto entre indivíduos afetados por problemas psicológicos.

Finalmente, destaca-se que a principal contribuição científica deste manuscrito está na apresentação de um instrumento válido e confiável para avaliar os sintomas de *burnout* em profissionais de enfermagem brasileiros, o qual possui arcabouço teórico robusto e atualizado, alinhado às recentes transformações ocorridas no mundo do trabalho em nível global.

Quanto às limitações deste estudo, ressalta-se a escassez de estudos de validação do BAT – Versão geral, o que impede uma análise aprofundada e a comparação dos resultados. Por outro lado, este fato reforça a originalidade e a relevância da presente investigação.

## Conclusão

O BAT – Versão geral demonstrou ser um instrumento robusto e consistente para avaliar sintomas de *burnout* em trabalhadores de enfermagem brasileiros. Neste sentido, o BAT – Versão geral pode se tornar uma importante ferramenta de gerenciamento para líderes de enfermagem e outros gestores de serviços de saúde que buscam implementar estratégias de promoção de ambientes de trabalho saudáveis, abrangendo aspectos físicos, psicológicos e emocionais da saúde dos trabalhadores.

Por fim, cabe ressaltar que os autores originais afirmam que o BAT não deve ser usado para o diagnóstico clínico de *burnout*. Em vez disso, representa um valioso instrumento de medida para ser utilizado na avaliação dos níveis individuais de sintomas de *burnout*.
